# An integrated approach to unravel the deep-shallow aquifer connectivity in the Eastern Sahara

**DOI:** 10.1038/s41598-026-38324-x

**Published:** 2026-03-02

**Authors:** Ibrahim A. Ibrahim, Abotalib Z. Abotalib, Haby S. Mohamed, Mahmoud M. Senosy

**Affiliations:** 1 Geology Department, Faculty of Science, Assiut University, Assiut, Egypt; 2 Division of Geological Applications and Mineral Resources, National Authority for Remote Sensing and Space Sciences, Cairo, Egypt; 3 National Center for Environmental Compliance, Riyadh, Saudi Arabia

**Keywords:** The Nubian aquifer system (NAS), Shallow aquifers, Connectivity dynamics, Stable isotopes, Sustainable development, Environmental sciences, Hydrology, Solid Earth sciences

## Abstract

The ambitious agricultural development projects in Egypt and the associated horizontal expansion into the core desert lands and desert fringe zones around the Nile Valley primarily depend on water availability. This study investigates the vertical recharge from the deep Nubian Aquifer System (NAS) toward the shallow aquifers, including the Carbonate and Quaternary aquifers in southern Egypt. While this connectivity has been studied locally through case studies, the present study integrates stable isotope data from all previous studies, together with analyses from vastly distributed new groundwater samples, remote sensing, and geophysical methods to better understand groundwater dynamics and aquifer connectivity over a regional domain. Findings show that: (1) the depths to the basement surface range from 350 to 4700 m below the land surface, (2) the major structural trends are E-W, NW–SE, NE-SW, ENE, NNW, and WNW trends, (3) the contribution ratios from the deep NAS to the overlying aquifers range between 10 and 98% as estimated using isotope mass balance calculations, and (4) the intersection of NW, ENE, and NE structural trends, which show similar trends between surface faults and deep faults, indicating vertical continuity, plays a major role in aquifer connectivity along the western desert fringes of the Nile River, particularly south of latitude 26°30′N. These findings indicate that the relatively thin sedimentary cover overlying the NAS south of latitude 26°30′N facilitates the upwelling of the NAS groundwater along with the intersection of the NW, ENE, and NE fault systems. Given the consensus of high hydraulic heads of the NAS compared to the overlying aquifers, the study suggests that a large-scale vertical upwelling at the deduced intersecting structural trends throughout the entire Limestone Plateau is worthy of further investigation. Such vertical upwelling could bring significant groundwater resources to shallow levels, as long as the NAS maintains its higher heads, and thus supports desert greening projects in Egypt. The findings also highlight the necessity of examining similar mechanisms in other desert environments with multiple aquifer systems.

## Introduction

Desert reclamation projects in Egypt are crucial for food security considering the escalating population growth during the last three decades^1^. These development projects involve the establishment of new agricultural and industrial settlements proximal to the Nile Valley in the easily accessible desert lands. In addition, the dramatic decrease of the fertile lands of the Nile Valley over the last few decades encourages farmers and small investors to reclaim new lands along the desert fringes^2^. The main issue that faces these ambitious development plans is the availability of water resources. The water supply for these projects is mainly obtained from the shallow aquifers, due to their easy accessibility^3^. However, the Egyptian deserts overlie some of the major aquifers, in terms of geographic extension and saturated thicknesses in Africa^4^, yet little efforts have been exerted to decipher the complexity of aquifer connectivity and groundwater dynamics in these desert areas. In this regard, understanding the connectivity between different aquifer systems is essential for sustainable water management, especially in regions with multiple aquifer systems and water scarcity issues.

**Fig. 1:**
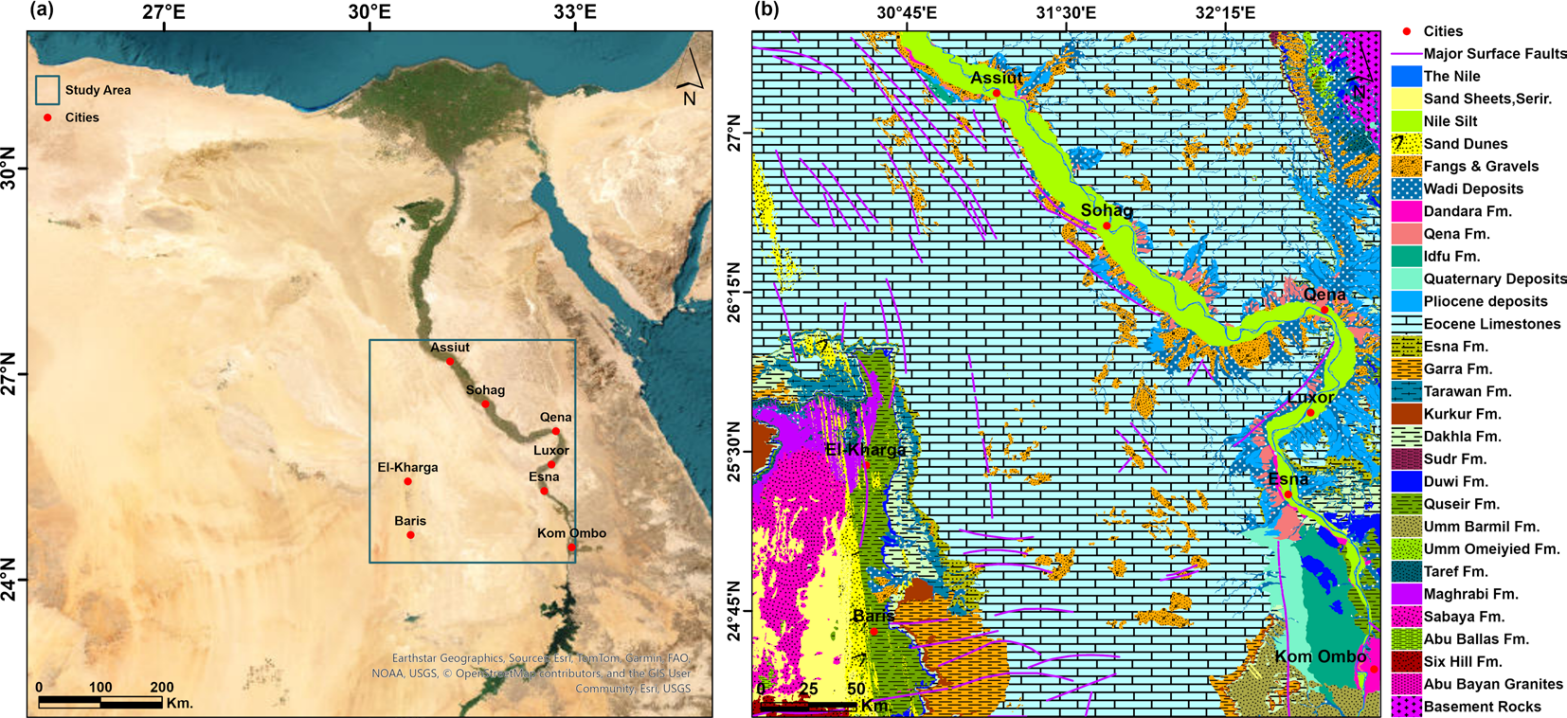
(**a**) Location map of the study area, and (**b**) Geological map of the study area after^37^, with major surface faults after EGSMA^69^. Created by ArcGIS Pro (version 3.5).

The Sahara Desert stretches across North Africa and represents the most expansive region on Earth with exceptionally elevated temperatures and low precipitation rates throughout the year^5^. The Sahara is distinguished by hyper-arid conditions at present at its core, having less than 50 mm of precipitation on average every year^6,7^. In this region, there are ongoing serious water scarcity problems with an anticipated water shortage of up to 90% of the present water budget by 2050^8,9^. The Southern Western Desert of Egypt and the Upper Nile Basin in Egypt represent the major geographic extent of the Eastern Sahara Desert. The study area stretches from the Nile Valley east to El-Kharga Oasis west; from 30° to 33°E, and from Aswan south to Assiut north; from 24.25° to 27.5°N, with a total surface area of 108,477 km^2^ standing for 10.8% of the total surface area of Egypt (Fig. 1a).

Besides the Nile River, the Eastern Sahara involves significant groundwater resources represented by major aquifers, including: (1) the transboundary Nubian Aquifer System (NAS) that represents the deep-water horizon, (2) the Carbonate Aquifer System (CAS), and (3) the Quaternary Aquifer System (QAS) that represent the shallow water horizons. The NAS is regarded as the major fossil aquifer system in North Africa with a spatial distribution of 2.2 million km^2^, shared between Egypt, Libya, Chad, and Sudan^10^. The NAS represents a vital and extensive groundwater source containing an estimated 150,000 km^3^ of groundwater^11^. However, lateral recharge can occur through occasional rainstorms, in addition to the Nile water, yet the vertical recharge between aquifers is vital in such desert core areas, particularly the zones located far away from the Nile River (i.e., up to hundreds of kilometers).

Vertical connectivity is achieved through vertical and sub-vertical fault systems of various scales, where the groundwater of the deep aquifer ascends through these features under pressure or high water head to feed the shallow aquifers^12^. Hence, it leads to providing a feeding source for the highly consumed shallow aquifers in arid regions^13^. For instance, the Great Artesian Basin (GAB) in Australia underlies approximately 1.7 million km^2^ of arid and semi-arid terrain. Structurally, the GAB is a multi-layered confined system featuring deep Jurassic and Cretaceous sandstones, similar to the NAS. While recharge occurs primarily laterally along its eastern margins, vertical upwards leakage is considered an important mechanism through intervening confining beds. This leakage results in flowing artesian springs along the arid discharge margins^14^.

The aquifer connectivity in the Eastern Sahara has been locally discussed in the literature^15–24^; however, these studies focused only on small-scale areas and have been limited to a restricted extension of the aquifers.

It was proven that during the Quaternary there were alternating periods of wet and dry climate conditions based on isotopic fractions of the analyzed groundwater samples^25,26^ in addition to the analysis of Quaternary sediments^27–29^. In this context, the wet periods represented periods of recharge for the fossil aquifers^29,30^, leading to high groundwater heads in the deep aquifers and allowing groundwater to rise through vertical/sub-vertical faults and mix with shallow water and ultimately discharge at the free surfaces, forming large-scale geomorphological features and depressions in the Eastern Sahara^17^.

According to reported isotopic data, the oxygen and hydrogen isotopic ratios of groundwater in northern Sudan are isotopically richer than those of groundwater from the Oases of Kharga, Dakhla, Farafra, and Bahariya in the Egyptian Western Desert^11,31–35^. This isotopic variability points out the mixing between the modern precipitation in northern Sudan, which is significantly enriched in isotopic composition, and the fossil groundwater of the NAS. In addition, it was demonstrated that the NAS receives an annual recharge from Lake Nasser estimated to reach 6.5 BCM/year at its highest level of 178 m amsl^10,34–36^. This makes the NAS a vital source to feed these shallow aquifers, even in the long-term water extraction strategies.

This present study employs an integrated approach utilizing remote sensing data, aeromagnetic data, deep borehole records, and isotopic analyses of groundwater samples to develop a conceptual model to better understand the regional groundwater dynamics and aquifer connectivity in the Eastern Sahara. This model will be achieved through the following steps: (1) analyzing various sets of satellite images to reveal the major surface faults and their trends, (2) processing and interpretation of aeromagnetic data in combination with borehole and Shuttle Radar Topography Mission (SRTM) data to qualitatively examine the trends of deep and shallow structural features and estimating the depth to basement, (3) determining the sources of recharge and the ratios of mixing between the aquifer systems using stable isotopic fractions, mainly (δD, δ^18^O) of collected groundwater samples and previously published isotopic data.

## Geological and hydrogeological settings

### Geologic settings

**Fig. 2:**
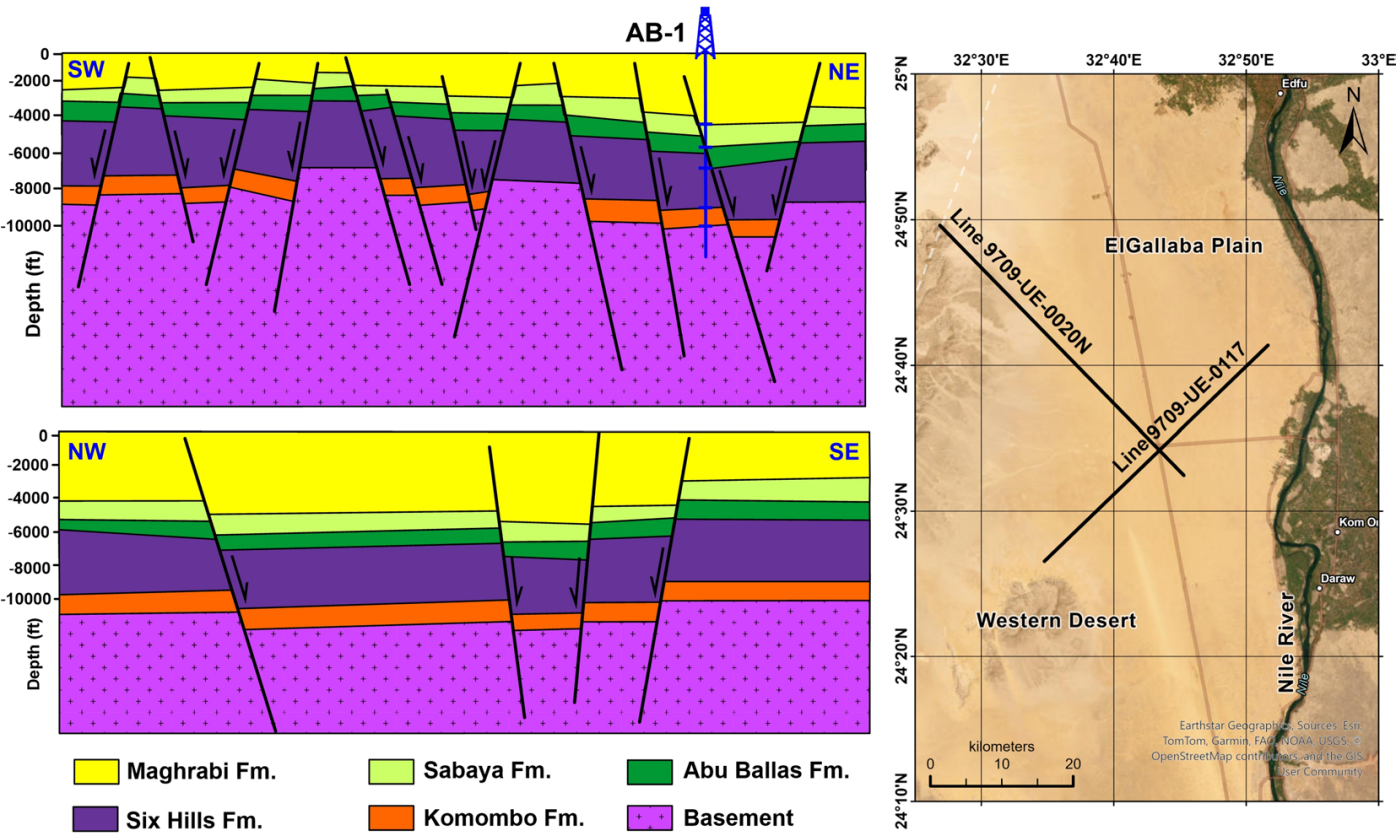
Subsurface cross-sections showing interpreted seismic sections of the geologic and structural settings of Komombo Basin. Adapted from: ‘Subsurface Structural Setting and Hydrocarbon Potentiality of the Komombo and Nuqra Basins, South Egypt: A Seismic and Petrophysical Integrated Study’, Abdeen, M. M., Ramadan, F. S., Nabawy, B. S., El Saadawy, O., *Natural Resources Research*, 30(5), Springer Nature, 2021, reproduced with permission from SNCSC. This figure is not part of the governing open access license of this publication but has been reproduced with permission. Created by ArcGIS Pro (version 3.5) and Golden Software Surfer (Version 29.3).

The sedimentary cover in the study area is reported from the geological map of Egypt^37^ (Fig. 1b). The study area includes the sedimentary successions ranging from the Lower–Upper Cretaceous succession of the Nubia Group sediments, followed by various successions ranging from the Campanian to the Ypresian in age and topped by the recent Quaternary sediments. The Nubia Group is the main unit of the NAS, namely the Six Hills, Abu Ballas, Sabaya, Maghrabi, and Taref formations, from older to younger, respectively^38^. Figure 2 illustrates this sequence with the major subsurface faults deduced from seismic data interpretation^39^.

The Nubia Group is topped by the shallow to deep marine Campanian-Lower Ypresian sequence represented by the Quseir, Duwi, Dakhla, Tarawan, and Esna formations and is composed mainly of shale, marl, and limestone^40^. This succession is followed conformably by the latest Ypresian predominantly carbonate succession known as the Thebes Formation^41^. The Quaternary sediments in the study area are represented by the Armant, Idfu, and Qena formations. These formations are composed of alternating beds of conglomerate, varied-size sands, and clay beds^42^.

### Hydrogeological settings

#### The Nubian aquifer system (NAS)

The NSAS is divided into two systems: (1) the deep Nubian Aquifer System (NAS) that consists of the Paleozoic and the Mesozoic deposits and tops the Precambrian basement complex directly^32^, and (2) the shallower Post Nubian Aquifer System (PNAS), located in the northern portion of the basin overlying the NAS, and it is made up of the Tertiary continental deposits^43^.

Three major subbasins form the NAS, namely, (1) the Kufra in Libya, northeastern Chad, and northwestern Sudan, (2) the Dakhla in Egypt, and (3) the Northern Sudan Platform subbasin in northern Sudan. The three basins are separated from each other by uplifts of Precambrian crystalline rocks^44^. The sedimentary sequence of the NSAS changes spatially with a maximum thickness of nearly 4, 3, and 0.5 km in the Kufra, Dakhla, and Northern Sudan Platform subbasins, respectively^32^. This aquifer system is characterized by high porosity (20%) and good hydraulic conductivity, ranging from 0.1 to 10 m/day^44^.

Measurements of radiochlorine (^36^Cl) and radiokrypton (^81^Kr) isotopes in deep groundwater from the northern regions of the NAS indicate a very long residence time of up to 0.2–1.3 × 10^6^ years^27,45^, and the groundwater age increases to the north except for areas proximal to the recharge areas, where reported ages do not exceed a few tens of thousands of years.

Groundwater heads in the NAS are high, reaching piezometric levels up to 149 m above mean sea level (msl) in the Western Desert, often described as having artesian conditions^17^. This hydraulic pressure contrasts sharply with the overlying shallow systems, where heads along the Nile Valley fringe typically range from 13 to 50 m above msl^2,15,18^. This large pressure difference drives a continuous ascending groundwater discharge^43^. Direct evidence of this deep hydraulic pressure is observed through: (1) groundwater mounding phenomena, where upwelling along faults creates local potentiometric highs that can rise up to 37 m above the average water table^43^, and (2) the existence of natural springs, such as sample 35 in this study, confirming localized artesian discharge to the surface where the deep head is sufficient to drive water without pumping.

#### The carbonate aquifer system (CAS)

This aquifer system is mostly karstified and fractured and is found in the Upper Cretaceous and Eocene limestone rocks, accounting for more than 50% of the outcrops of Egypt. This aquifer occupies the northern and middle portions of the Western Desert, indigenously in the Eastern Desert, and is in the northern and central Sinai Peninsula^46^. This karstic aquifer is characterized by the development of significant secondary porosity through numerous fracture networks^47^.

#### The quaternary aquifer system (QAS)

The QAS in Egypt designates a granular aquifer containing water from the Pleistocene and Holocene epochs, which refers to the Nile Valley and Nile Delta aquifers. These two aquifers supply about 87% of the used groundwater in Egypt for irrigation and domestic purposes, given the high extraction rates of more than 7 BCM/year^9^ and the significantly low economic accessibility costs. The QAS is regarded as one of the most important aquifer systems in Egypt. The Nile branches, channels, and the percolating irrigation water are the principal sources of recharge for this aquifer^48^.

The Nile Valley aquifer consists of two hydrogeological zones^49,50^. The lower one is composed of Pleistocene permeable sands and gravels, forming the base with high hydraulic conductivity ranging from 60 to 100 m/day^21^. This aquifer is the main and dominant unit of the QAS in Egypt. The upper unit is a Holocene semi-permeable clay-silt layer with low horizontal and vertical permeability, which acts as an aquitard^19,51^. The groundwater level depths range from 3 to 20 m in the floodplain of the valley^49,52^.

^19^ considered the concentrations of tritium in groundwater as indicators of groundwater age for samples at the rim of the Nile Valley, including the QAS in Sohag area. Tritium and stable isotope analyses revealed that the groundwater was recharged from the Nile before the tritium peak in the early sixties^53^ and before the establishment of the Aswan High Dam in 1971. However, groundwater near the River Nile has a similar isotopic signature close to that of the Nile water, implying that recent recharge occurs from the present Nile water.

## Data acquisition and preparation

### Remote sensing data

The Landsat-8 false color composite mosaic was used in this study to create a basemap (acquisition date: 2017; composed of 4 scenes with a spatial resolution of 30 m) and to delineate surface faults. Moreover, a mosaic of several Shuttle Radar Topography Mission (SRTM) images was utilized to produce a painted hillshade mosaic (acquisition date: 2000; composed of 32 scenes with a spatial resolution of 30 m) and to unravel faults and shear zones hidden by thin (less than 1 m) aeolian sediment beds^54^. Additionally, to validate field-based and orbital fault mapping, high-resolution satellite imagery was used (Available via ArcGIS online basemaps by ESRI). To prevent any unwanted dimensional distortion across the different data sets, the acquired data were referred to a uniform projection (Datum; WGS 84: Universal Transverse Mercator (UTM) zone; 36N) within a GIS workspace.

### Aeromagnetic data

The used aeromagnetic data are reduced-to-pole (RTP) two aeromagnetic data sheets (sheet 5 and sheet 8) with a scale of 1:500,000 published in 1989 by the Egyptian General Petroleum Corporation (EGPC) with a twenty-five nT contour interval. The total magnetic intensity anomaly data was converted to the pole using mean inclinations and declinations of the geomagnetic field of 39.5°N, 2.0°E and 37.7°N, 2.0°E for both of the sheets, respectively. Reduction to the pole was first introduced by^55^ to remove the shift of the anomalies and make it localized over the source because of magnetic latitude and dip magnetization. To ensure high spatial fidelity for the regional structural analysis, the aeromagnetic maps (scale 1:500,000) were digitized through systematic contour digitization. Individual contour lines (25 nT interval) were digitized as high-density polygons and polylines using Didger 5 software. Georeferencing was executed using the map’s original coordinate crosses, achieving a Root Mean Square Error (RMSE) of less than 1.25 m. This level of accuracy ensures that the digitized dataset provides a robust and spatially precise foundation for the subsequent filtering and modeling processes.

These vectors were subsequently used to generate an RTP magnetic grid via the Ordinary Kriging interpolation method using a 100 × 99 m grid cell size by Surfer 25 software. This interpolation technique was selected as it provides the best linear unbiased estimate data, relying on the critical assumption of intrinsic stationarity and minimizing interpolation variance based on the spatial autocorrelation derived from the empirical variogram. The gridded data were exported in a csv file format to be used directly in Geosoft Oasis montaj 8.4 software. Before the processing of the RTP anomaly map, the upward continuation to 3000 m was conducted as an introductory procedure. This is to eliminate the noise and overcome the ridging problem caused by very high frequency^56^. After several iterations in the interactive spectral filtering panel in oasis montaj and by comparison to the original RTP map, the 3000-m upward continuation distance was selected. For this data, distances less than this value would not decompose the noise well, while a higher value would alter the magnetic bodies’ shape and magnitude.

### Groundwater field sampling

**Fig. 3:**
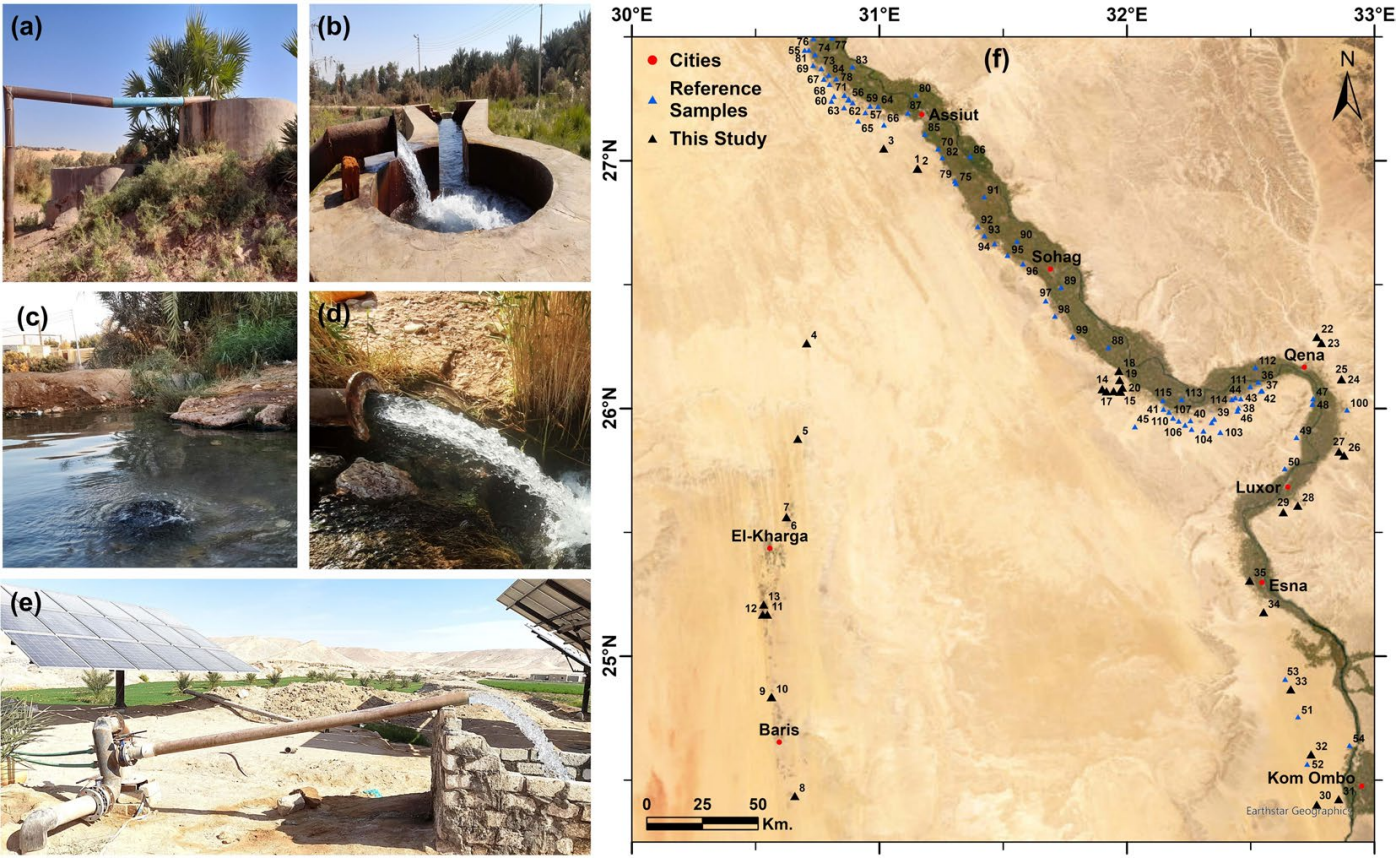
Field photos for (**a**) Sample 11, (**b**) Sample 13, (**c**) Sample 23, (**d**) Sample 25, (**e**) Sample 35, and (**f**) the locations of the samples collected during this study and the reference samples. Created by ArcGIS Pro (version 3.5).

A field trip was conducted in December 2022, where thirty-five groundwater samples were collected from the available groundwater wells in the study area for the stable isotopic analyses. The locations of the wells were chosen to cover the study area (Fig. 3). The sampling started from Assiut Governorate to the north of Aswan, and the second destination was El-Kharga Oasis. Ten samples were collected from El-Kharga Oasis, while the other twenty-five samples were collected within and around the Nile Valley (Table 1). The wells have depths ranging from 45 to 705 m, and the depth to groundwater (DGW) ranges from 10 to 120 m. All these wells are used for irrigation purposes, except well 10A, which is used for domestic purposes with a total depth of 435 m. Samples 23 and 35 were collected from springs.

**Table 1:** Showing the data and the isotopic composition (δ^18^O, δD) of the analyzed water samples.

NO	DGW (m)	TWD (m)	TDS (ppm)	δ^18^O (‰)	δD (‰)	Tapping Aquifer
1	10	80	1240	− 10.48	− 77.05	CAS
2	14	160	820	− 11.73	− 80.84	CAS
3	50	600	400	− 12.06	− 84.03	CAS
4	50	450	547	− 11.56	− 83.82	NAS
5	65	250	572	− 10.85	− 81.28	NAS
6	55	673	450	− 11.46	− 88.26	NAS
7	45	496	442	− 12.33	− 89.4	NAS
8	55	705	350	− 11.99	− 88.78	NAS
9	45	435	750	− 12.09	− 87.84	NAS
10	45	430	850	− 11.55	− 86.95	NAS
11	-	Deep	563	24.23	3.83	NAS
12	-	Deep	484	− 2.96	− 0.76	NAS
13	−	Deep	572	− 0.63	− 0.83	NAS
14	95	145	1060	− 10.81	− 79.96	CAS
15	50	75	1270	− 9	− 65.43	QAS
16	75	180	1110	− 10.05	− 71.27	QAS
17	90	160	1110	− 11.36	− 79.74	QAS
18	14	50	1600	1.8	9.98	QAS
19	30	45	1870	− 7.89	− 56.32	QAS
20	48	80	1360	− 7.97	− 59.35	QAS
21	55	110	1220	− 8.56	− 63.65	QAS
22	35	70	3600	− 5.53	− 37.2	QAS
23	0	−	1695	− 7.77	− 49.99	Artesian
24	75	90	3100	− 3.59	− 25.76	QAS
25	80	90	2630	− 1.78	− 20.5	QAS
26	20	80	3730	− 6.21	− 59.74	QAS
27	35	60	2880	− 2.4	− 28.69	QAS
28	40	150	3320	− 3.46	− 32.04	QAS
29	65	114	1050	− 1.06	− 12.79	QAS
30	120	198	1000	− 9.84	− 71.76	CAS
31	120	180	1800	− 8.03	− 70.79	CAS
32	50	220	1760	− 9.89	− 75.15	CAS
33	65	188	1620	− 1.51	− 19.95	CAS
34	40	96	1540	− 7.34	− 56.02	QAS
35	0	−	2140	− 9.35	− 67.43	Artesian

All samples were collected directly from the wells and springs; sampling included at least a 30-min purge to ensure good representativeness of the in-situ groundwater. The samples were collected in high-density 30 ml polyethylene bottles, and the standard criteria for sampling and preservation were ensured. During the fieldwork, the samples were preserved in ice-filled conditioned insulating foam boxes to keep the samples away from direct sun heat.

## Study methodology

The study area encompasses 108,477 km^2^ which represents approximately 10.8 percent of Egypt’s total surface area. To ensure representative characterization across this extensive region, we utilized a hierarchical integrated approach where regional-scale data layers, specifically satellite imagery and aeromagnetic grids, provide continuous spatial coverage and mapping of the structural framework. The groundwater sampling for 35 water wells was strategically targeted to capture the hydrogeological feeding patterns. This methodology is consistent with standards for assessing aquifer dynamics in vast, data-scarce arid mega aquifer systems, such as the Great Artesian Basin^14^. In these studies, discrete geochemical and isotopic data serve as critical validation for the broader flow dynamics established by regional geophysical models that are usually used as a proxy for data-scarce or data-absent areas.

**Fig. 4:**
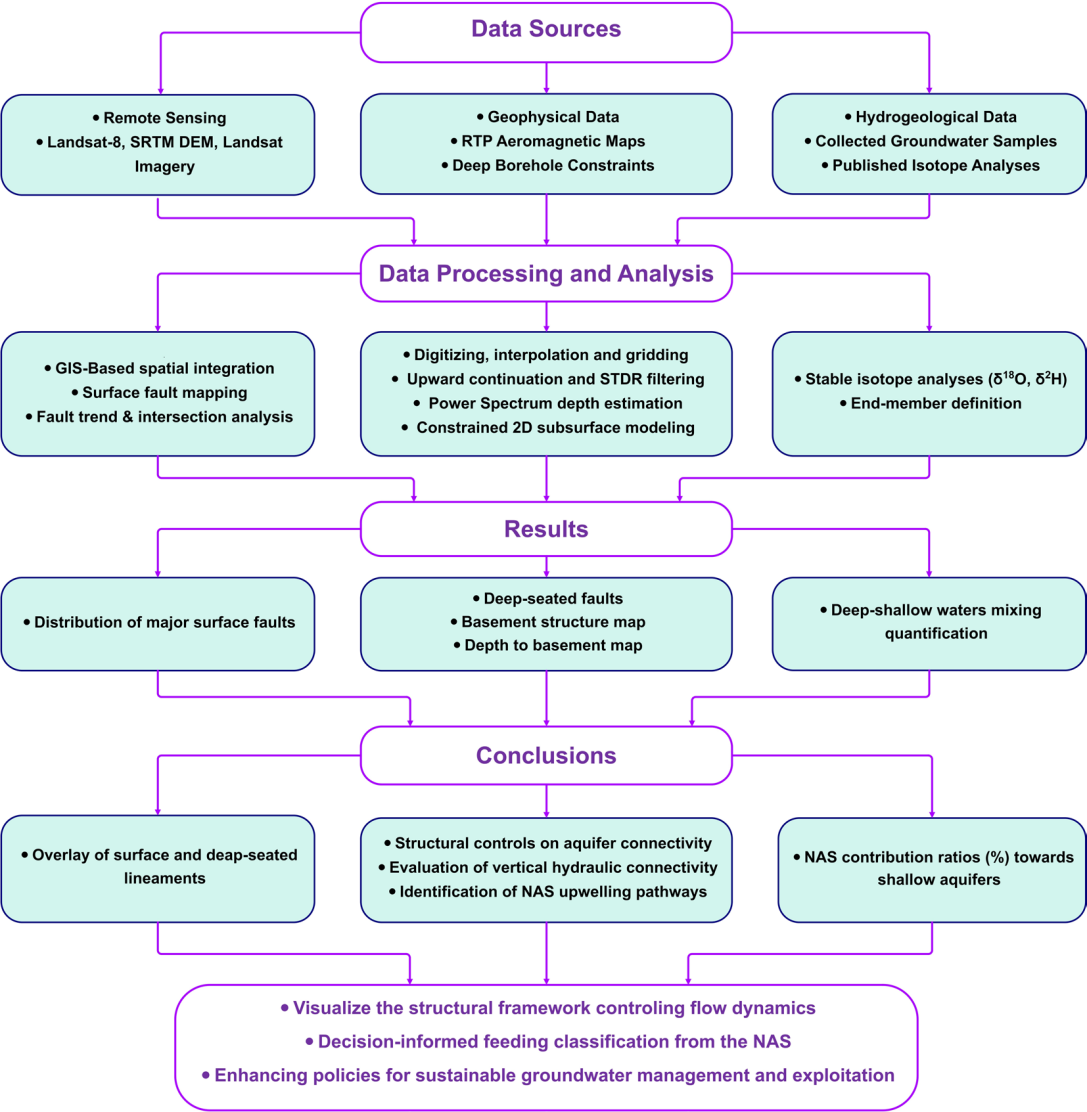
A schematic flowchart showing the methodology implemented in the current study.

The implemented methodology in the current study is summarized in a flowchart (Fig. 4) including the integrated remote sensing, geological, geophysical and stable isotopes datasets in addition to the applied procedures to process and interpret these datasets.

The remote sensing data in this study are utilized to reveal the major surface faults over the study area. Aeromagnetic data in conjunction with deep borehole data, SRTM data, and various geological materials were utilized to unravel the subsurface structural environment controlling the study area. In addition, these data were used to create the basement-relief and depth-to-basement maps to evaluate the role of the Precambrian basement rocks in connecting the different aquifer systems in the study area. The surface faults revealed from remote sensing data are analyzed in comparison to the deep-seated major faults deduced from aeromagnetic data to examine the upward continuity of these structures. In addition, stable isotope analyses (δD, δ^18^O) are used to reveal the isotopic signatures of the groundwater samples. This integrated methodology can visualize the surface and subsurface structural frameworks, identify and estimate feeding sources towards the shallow aquifers. The following sections show the applied techniques to achieve the study methodology.

### Second-order tilt derivative (STDR)

The tilt derivative filter (TDR) is commonly used as it does an excellent job of mapping subsurface structures, without the noise amplification that may occur in higher-order vertical derivative grids^57,58^. The tilt derivative is specified according to Eq. 1:

1 $${\mathrm{TDR}} = {\mathrm{tan}}^{ - 1} \left( {\frac{{{\mathrm{FVD}}}}{{{\mathrm{THD}}}}} \right)$$

where FVD is the first vertical derivative defined in (Eq. 2) and THD is the total horizontal derivative of the total magnetic field F (Eq. 3), where:

2 $${\mathrm{FVD}} = \frac{{\partial {\mathrm{f}}}}{{\partial {\mathrm{z}}}}$$

3 $${\mathrm{THD}} = \sqrt {\left( {\frac{{\partial {\mathrm{f}}}}{{\partial {\mathrm{x}}}}} \right)^{2} + \left( {\frac{{\partial {\mathrm{f}}}}{{\partial {\mathrm{y}}}}} \right)^{2} }$$

The FVD is used to bring out the hidden local variation in the potential field data that could not be detected by the regional-residual separations, and to improve the clearance of geological contacts^59^. The THD is employed to identify contact patterns and determine boundaries of susceptibility contrasts in magnetic data over basement surfaces. A key advantage of the THD is its reduced sensitivity to noise, as it only involves computing the two first-order horizontal derivatives of the magnetic field^60^. In comparison to the FVD method, which is effective for detecting shallow structures, the THD method excels at delineating both shallow and deep magnetic sources.

^61^ presented an improved TDR filter utilizing the proportion of the second-order vertical derivative (SVD) to the second-order THD in the tilt angle equation, named second-order tilt derivative (STDR) (Eq. 4), where:

4 $${\mathrm{STDR}} = {\mathrm{tan}}^{ - 1} \left( {{\mathrm{M}} \cdot \frac{{\frac{{\partial^{2} {\mathrm{f}}}}{{\partial {\mathrm{z}}^{2} }}}}{{\sqrt {\left( {\frac{{\partial {\mathrm{THD}}}}{{\partial {\mathrm{x}}}}} \right)^{2} + \left( {\frac{{\partial {\mathrm{THD}}}}{{\partial {\mathrm{y}}}}} \right)^{2} } }}} \right)$$

The SVD improves the delineation of contacts and boundaries of magnetic sources, making it useful in structural interpretation. The maxima and minima of the STDR define the positive and negative anomalies, respectively. The STDR was computed using Fast Fourier Transform (FFT) operators in the wavenumber domain by the Grid Math option in Oasis montaj package according to Eq. 4. The derivatives were calculated directly on the upwardly continued gridded dataset which was derived via Ordinary Kriging Interpolation of the RTP maps.

### Power spectrum (PS) technique

The Power Spectrum (PS) technique was devised by^62^, it is a method used to compute the depth of magnetic sources relying on a statistical basis. This statistical aspect transforms the data from the spatial to the frequency domain using a FFT operator. This transformation is to allow the analysis of the data frequency components. The power spectrum of the transformed data is calculated, which represents the distribution of power or energy of the magnetic field as a function of frequency. This spectrum is plotted as a curve that displays linear segments; each segment corresponds to the depth of a set of magnetic sources. By calculating the slope of each segment, the method allows for estimating the depths of these concealed bodies using Eq. 5.

5 $${\mathrm{Z}} = \frac{{ - {\mathrm{Slope}}}}{{4{\uppi }}}{ }$$

### 2D profiling and basement maps

2D subsurface modeling is a geophysical technique used to create a theoretical model of subsurface geological structures in two dimensions and calculate the expected geophysical responses that would be observed at the surface^63^. 2D subsurface modeling in this study is performed with the interactive GM-SYS modeling tool, part of the 2015 Geosoft Oasis montaj software suite. Subsurface information of 18 deep wells reaching the basement surface (Table 2) was used as constraints for controlling the obtained 2D models. Thirty 2D profiles have been constructed and distributed to pass through most major, moderate, and minor anomalies. This is to cover the entire study area and the changes in the basement topography and infer the subsurface structural controls as much as possible. At first, thirteen profiles were modeled in an even distribution and trending mainly from the west to east. To overcome the problem of absent basement penetrating wells in some areas, four profiles, PP’14, 15, 16, and 17, along which deep wells are present, have been modeled. The intersection points between these profiles and the profiles that lack well data were used as control points. Thirteen control points were used to model the profiles PP’18 to PP’30; the data of the deduced points are shown in Table 2. The east–west trending profiles are 305 km in length for each, while profiles PP’14, 15, 16, and 17 are 308, 311, 340, and 235 km, respectively.

**Table 2:** Shows the deep wells reaching the basement (ID = BH), and control points deduced from constructed profiles (ID = CP) to construct basement maps.

ID	well name	Depth (m)	ID	Well name	Depth (m)
BH-1	Baris 15	425	BH-17	El-Balyana	1632
BH-2	Baris 14	527	BH-18	Rewedea	2200
BH-3	Komombo-3	1222	CP-1	–	1950
BH-4	Baris 3	429	CP-2	–	1860
BH-5	AB-1	2609	CP-3	–	980
BH-6	Baris 7	733	CP-4	–	1090
BH-7	Mem-1	1515	CP-5	–	1400
BH-8	Baris 11	654	CP-6	–	1500
BH-9	SG-6	1000	CP-7	–	1750
BH-10	Gurmashin 5	802	CP-8	–	1980
BH-11	SG-9	975	CP-9	–	3200
BH-12	Bulaq 10	718	CP-10	–	4000
BH-13	Bulaq 5	770	CP-11	–	3050
BH-14	Ginah 11	836	CP-12	–	2800
BH-15	Ginah 12	700	CP-13	–	1400
BH-16	Kharga West 2	707			

To develop a geological model where the calculated magnetic effect should align with the observed magnetic anomaly profile, each polygon, representing a structural or lithological feature, is assigned a specific susceptibility value. The susceptibility values for sedimentary rocks are typically extremely low compared to those for igneous rocks, which can often be considered negligible in regional studies. The basement rocks in the modeled cross-sections have susceptibility values ranging from 0.002 to 0.048 SI units^64^ consistent with the advocated subtle basement rock types in the Western Desert in Egypt. In addition, these values were validated by the blocks under each borehole where the depth of the basement rocks is actually known and the susceptibility can be inferred. The bottom surface for the basement blocks is constructed horizontally at 8 km depth and unified for the thirty profiles. Through manual iterations, adjustments were made to each structural unit or block, and susceptibility values were updated. After modeling these cross-sections, their numerical data were combined, interpolated, and gridded to obtain the basement relief map related to the zero level above msl. DEM data of the study area was gridded from the SRTM data to form the topography surface of the area; this is to calculate and obtain the thickness of the sedimentary succession.

### Groundwater samples analysis

Groundwater samples were analyzed for the stable isotopic composition (δ^18^O, δD) using the ABB LGR-laser isotope analyser in the Division of Geological Applications and Mineral Resources, National Authority of Remote Sensing and Space Sciences, Cairo, Egypt. The measurements are expressed in terms of delta notation (δ) in per mille (‰) deviation units relative to the Vienna Standard Mean Ocean Water (V-SMOW), described by Eq. (6),

6 $${\updelta }\left( {{\mathrm{sample}}} \right) = \left( {\frac{{{\mathrm{R}}_{{{\mathrm{sample}}}} }}{{{\mathrm{R}}_{{{\mathrm{standard}}}} }}} \right) - 1 * 10^{3}$$

where R = ^18^O/^16^O or ^2^H/^1^H. Typical accuracy is nearly ± 1.5‰ and ± 0.1‰ at 1 standard deviation for ^2^H and ^18^O, respectively. For comparison purposes, the collected samples were correlated with previously published isotopic data^15–21. In addition, the previously published isotopic data^ to provide a large-scale overview of the groundwater isotopic signature in the Eastern Sahara.

### Evaluation of groundwater mixing fractions

To quantify the mixing ratios between the two end-members (recent and Pleistocene-recharged water), Eq. 7 was used:

7 $${\updelta }_{{\mathrm{t}}} = ({\updelta }_{1} \times {\mathrm{X}}) + \left( {{\updelta }_{2} \times \left[ {1 - {\mathrm{X}}} \right]} \right)$$

and rearranging Eq. 7 for simplification, it yields Eq. 8:

8 $${\mathrm{X}}\left( {\mathrm{\%}} \right) = \frac{{{\updelta }_{{\mathrm{t}}} - {\updelta }_{2} }}{{{\updelta }_{1} - {\updelta }_{2} }} \times 100$$

where δ_t_ is the δ^18^O value of the measured δ^18^O of the groundwater samples; δ_1_ is the δ^18^O value of the deep-aquifer isotopic end-member (NAS depleted isotopic signatures in this study), and δ_2_ is the δ^18^O value of the shallow-aquifer isotopic end-member (shallow aquifers enriched isotopic signatures in this study). According to this terminology, X is the contribution ratio in percentage from the deep NAS to the shallow aquifers in the groundwater samples, respectively.

Equation 8 was used for δ^18^O, regarding an end-member for the NAS fossil water in the Kharga Oasis^27,65,66^, calculated as the arithmetic mean for these samples, equal to − 11.61‰ for δ^18^O, and an end-member for the Nile water, equal to -0.6‰ for δ^18^O^19,67^.

## Results

### Mapping surface faults using remote sensing datasets

The surface faults were traced and mapped, depending on the geomorphological characteristics provided by^68^. These characteristics include: (1) Displacement of rock beds, which is visible on the surface as a discontinuity in the bedding planes, (2) Scarps where fault scarps are steep slopes or cliffs formed due to the vertical displacement along a fault, and (3) Changes in drainage patterns as abrupt changes in the course of streams and rivers. The surface faults mapped from the geological map of Egypt^69^ are used to verify the obtained fault trends deduced from the remote sensing datasets.

**Fig. 5:**
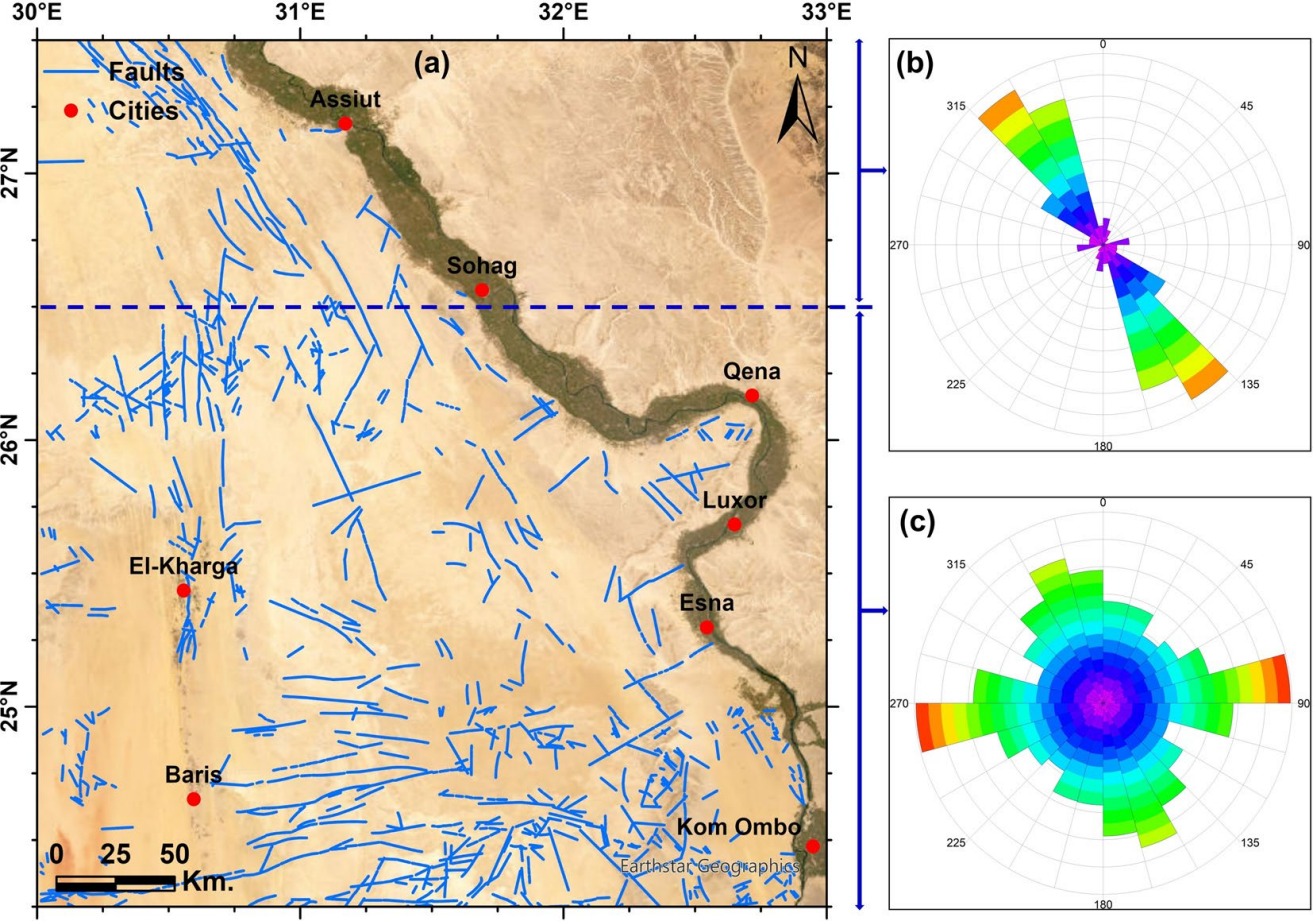
(**a**) Major surface faults over the study area mapped from remote sensing datasets, rose diagram for the surface faults for (**b**) the northern part (**c**) the southern and middle parts. Created by ArcGIS Pro (V 3.5).

With the aid of the empty desert environment in the study area, we obtained the surface faults on the western side of the Nile Valley (Fig. 5a). The fault trends deduced from the remote sensing data show that the NW–SE and NNW trends prevail in the northern part of the study area (Fig. 5b). While various tectonic trends, the E-W, NNW, NW–SE, ENE, and NE-SW trends, prevail in the southern and middle parts (Fig. 5c).

### Mapping subsurface faults and basement relief

#### Reduced to magnetic pole (RTP) map

**Fig. 6:**
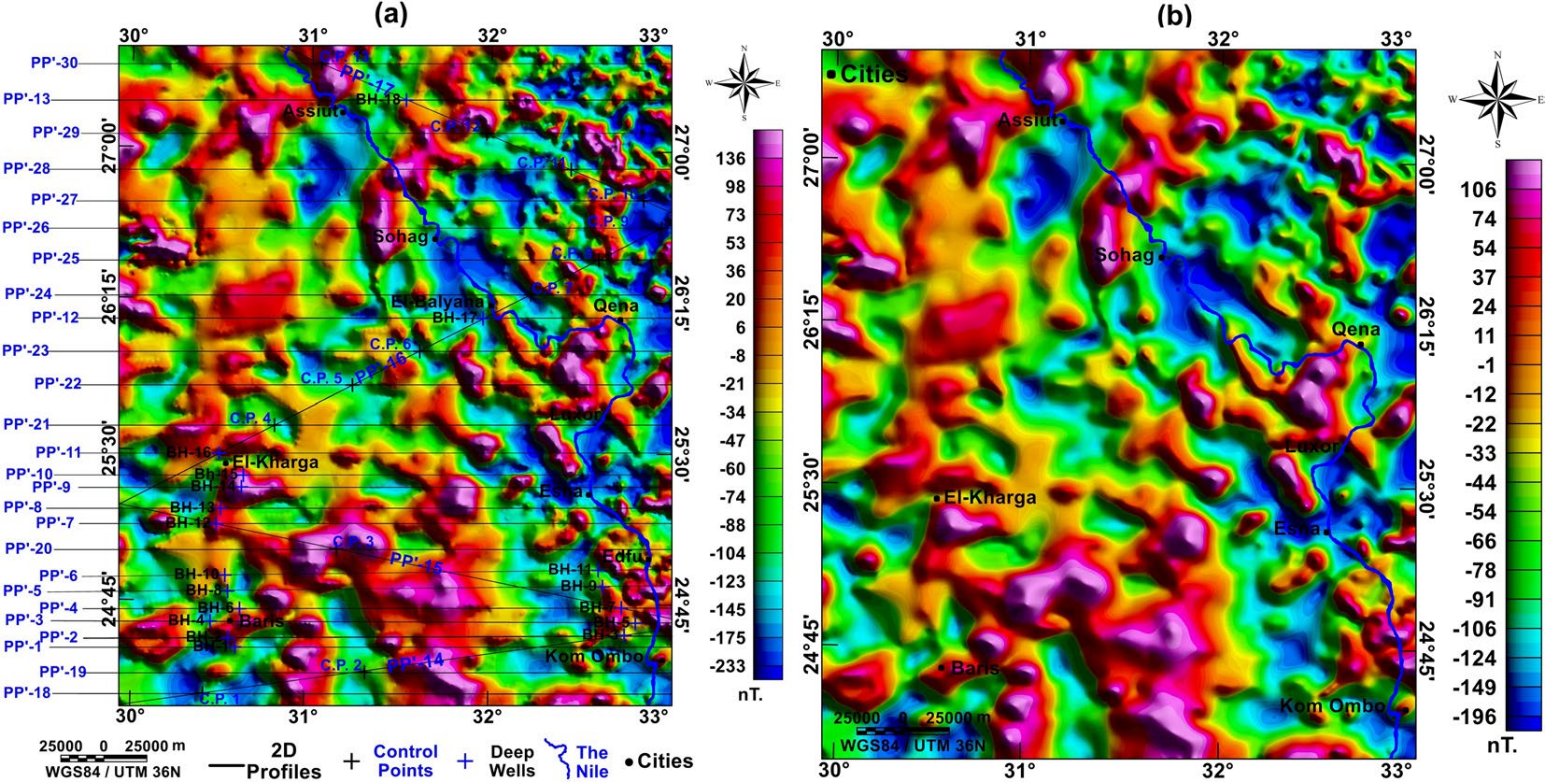
(**a**) RTP map with the 2D profiles, locations of deep boreholes and control points, and (**b**) upward continuation map to 3000 m for the RTP map. Created by Geosoft Oasis montaj (V 8.4).

Figure 6a represents the obtained RTP map of the study area, the designed profiles to construct the 2D models, and the location of the deep wells. The high, red-shaded magnetic anomalies are intermittent through the study area; their magnitudes range from 45 to 150 nT. On the other hand, the low magnetic anomalies with blue shade range from -200 to 150 nT. For large-scale magnetic data in this study, this variation could be related to different basement depths from an area to another and variation of the susceptibility values for different basement rock types. The southern part of the map has higher values than the northern part of the map, meaning that the northern part is deeper than the southern and middle parts of the study area. The resulting upward continuation map preserved anomaly geometry and does not distort or eliminate any of the anomalies; it only decomposes the very high-frequency noise, and it enhances the original anomalies (Fig. 6b).

#### Second-order tilt derivative (STDR) map

**Fig. 7:**
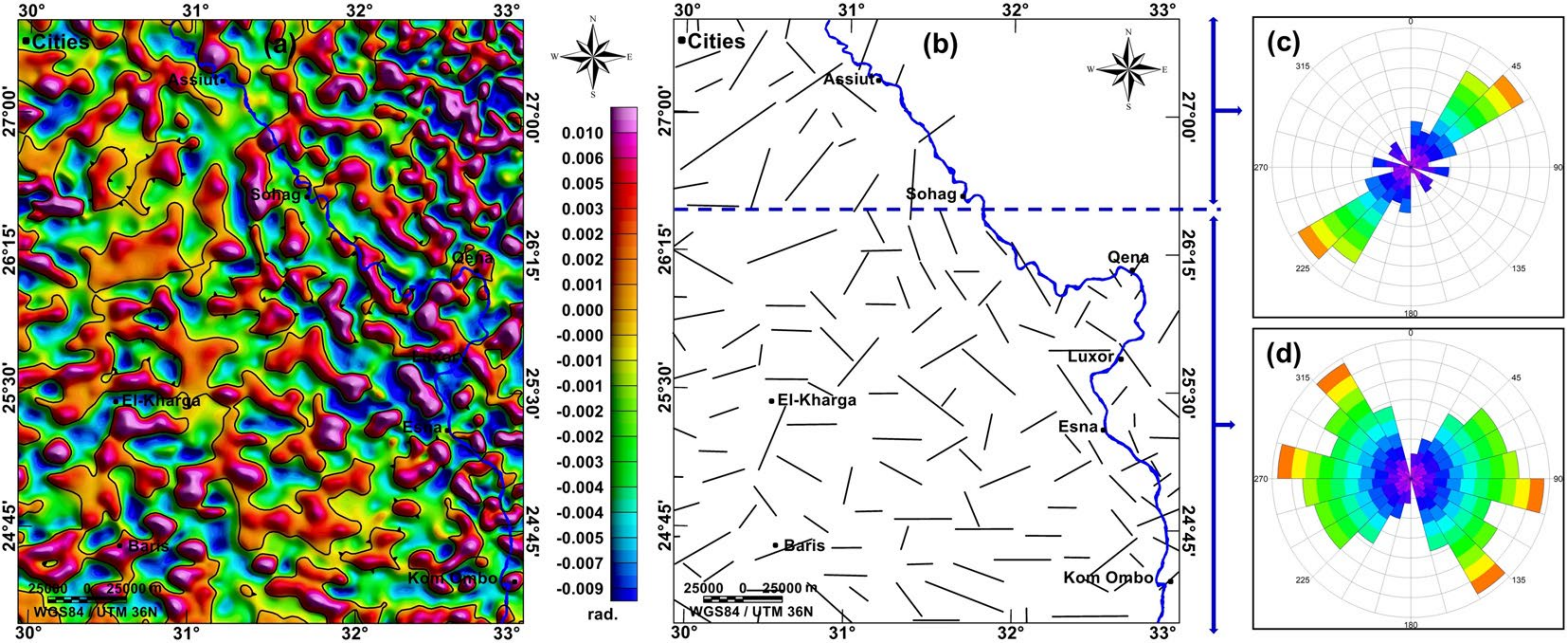
(**a**) STDR map of aeromagnetic data with a zero-line contour (**b**) subsurface major faults mapped from aeromagnetic data, rose diagram for the mapped faults in (**c**) the northern part, and (**d**) in the southern and middle parts. Created by Geosoft Oasis montaj (V 8.4).

The STDR filter (Fig. 7a) delineated the bodies’ edges in an efficient way, where the contouring represents the zero-contour line marking the rims of these bodies, and the maxima represent the stretch of the subsurface faults (Fig. 7b). The analysis of the subsurface fault trends yielded that the NE-SW trend is prevailing in northern part of the study area (Fig. 7c), while the E-W, NW–SE, WNW, NE-SW, NNW and ENE are prevailing the southern and middle parts of the study area (Fig. 7d). While the RTP map gives a rapid qualitative interpretation of the subsurface distribution of magnetic properties, the STDR succeeded in delineating the edges of the subsurface magnetic anomalies that could be interpreted as faults.

#### Power spectrum (PS) curve

**Fig. 8:**
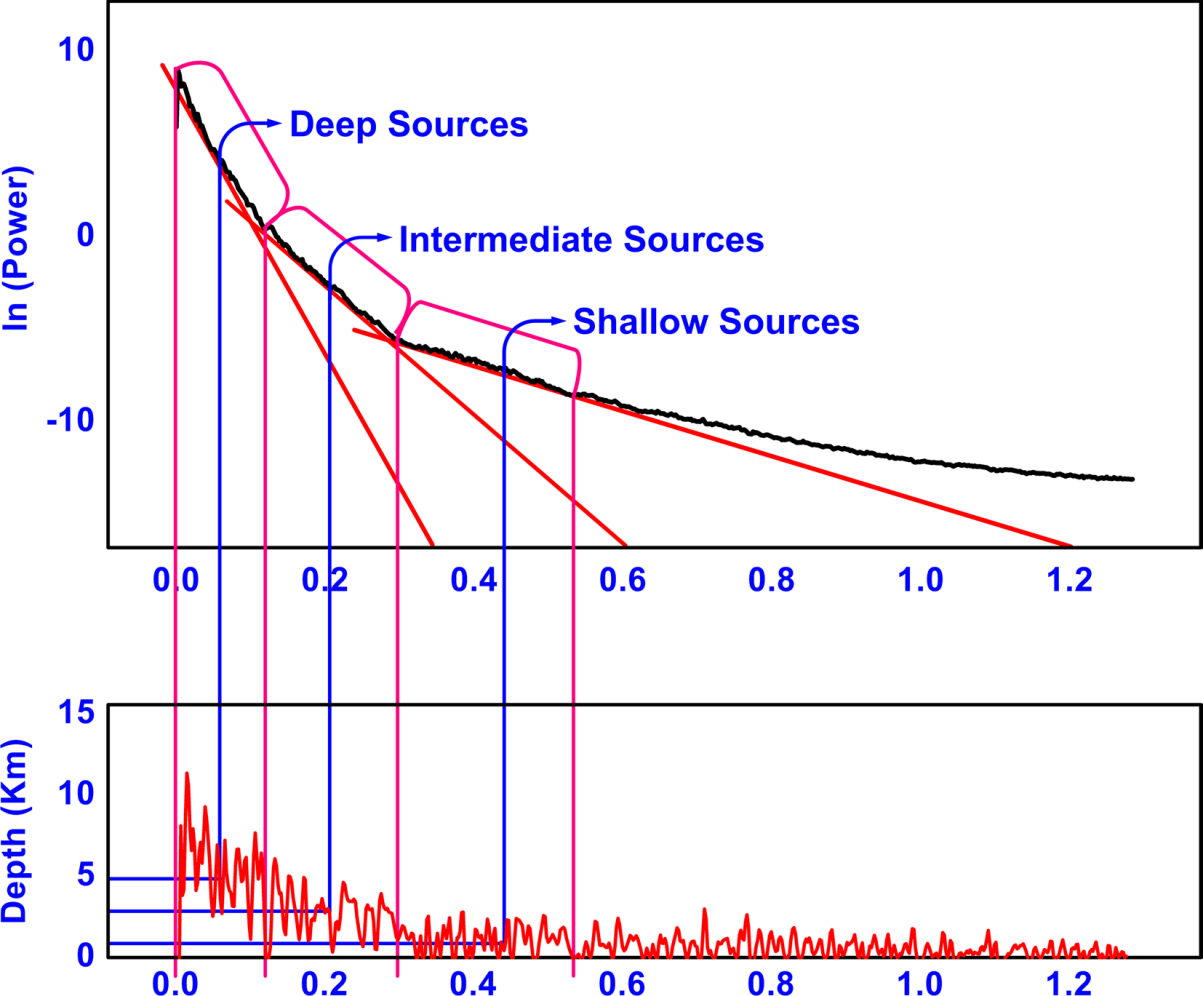
A 2D Power Spectrum of the aeromagnetic data.

A 2D Power Spectrum (PS) curve was obtained (Fig. 8). By calculating the slope of each segment and imposing its value in Eq. 6, it yielded the average depth for the deep, intermediate, and shallow magnetic bodies as 5.3, 2.6, and 0.8 Kilometers, respectively. The data obtained from the PS curve are summarized in Table 3.

**Table 3:** Shows the depths and wavenumber ranges of magnetic bodies in the study area deduced by Power Spectrum (PS) technique.

Magnetic bodies	Wavenumber (/km)	Depth (km)
Deep	0–0.12	5.3
Intermediate	0.12–0.3	2.6
Shallow	0.3–0.53	0.8

#### 2D-profiling and basement maps

**Fig. 9:**
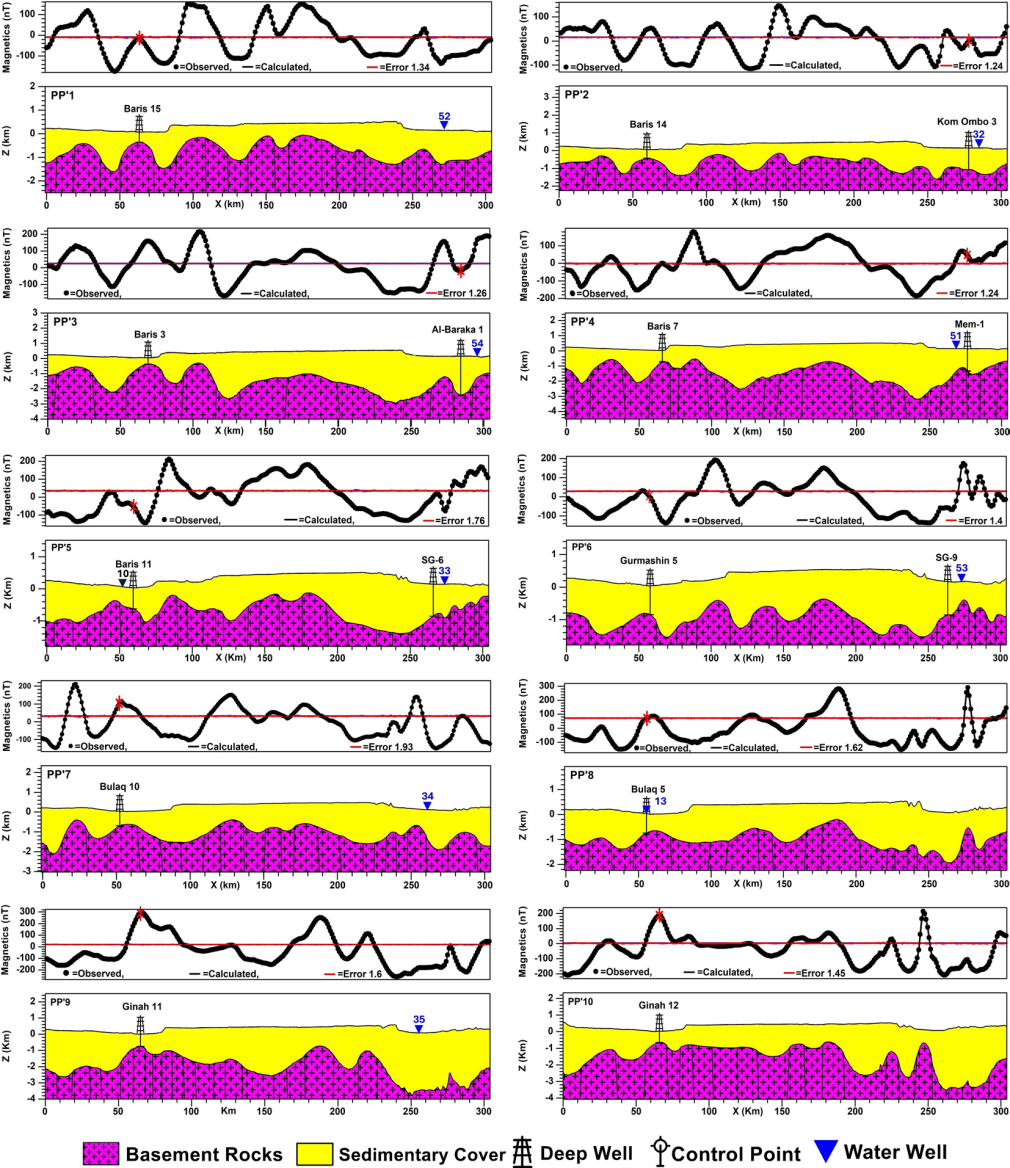

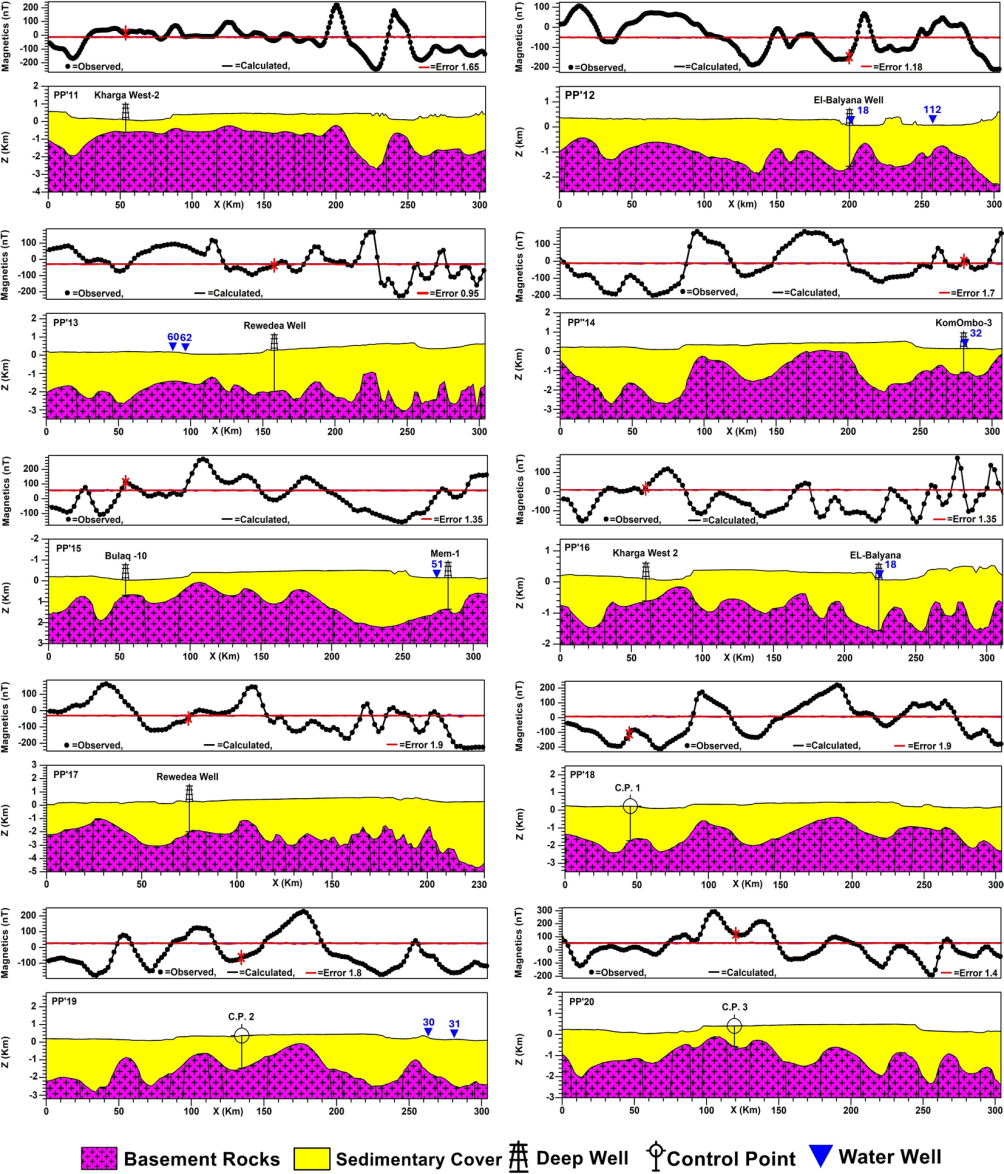

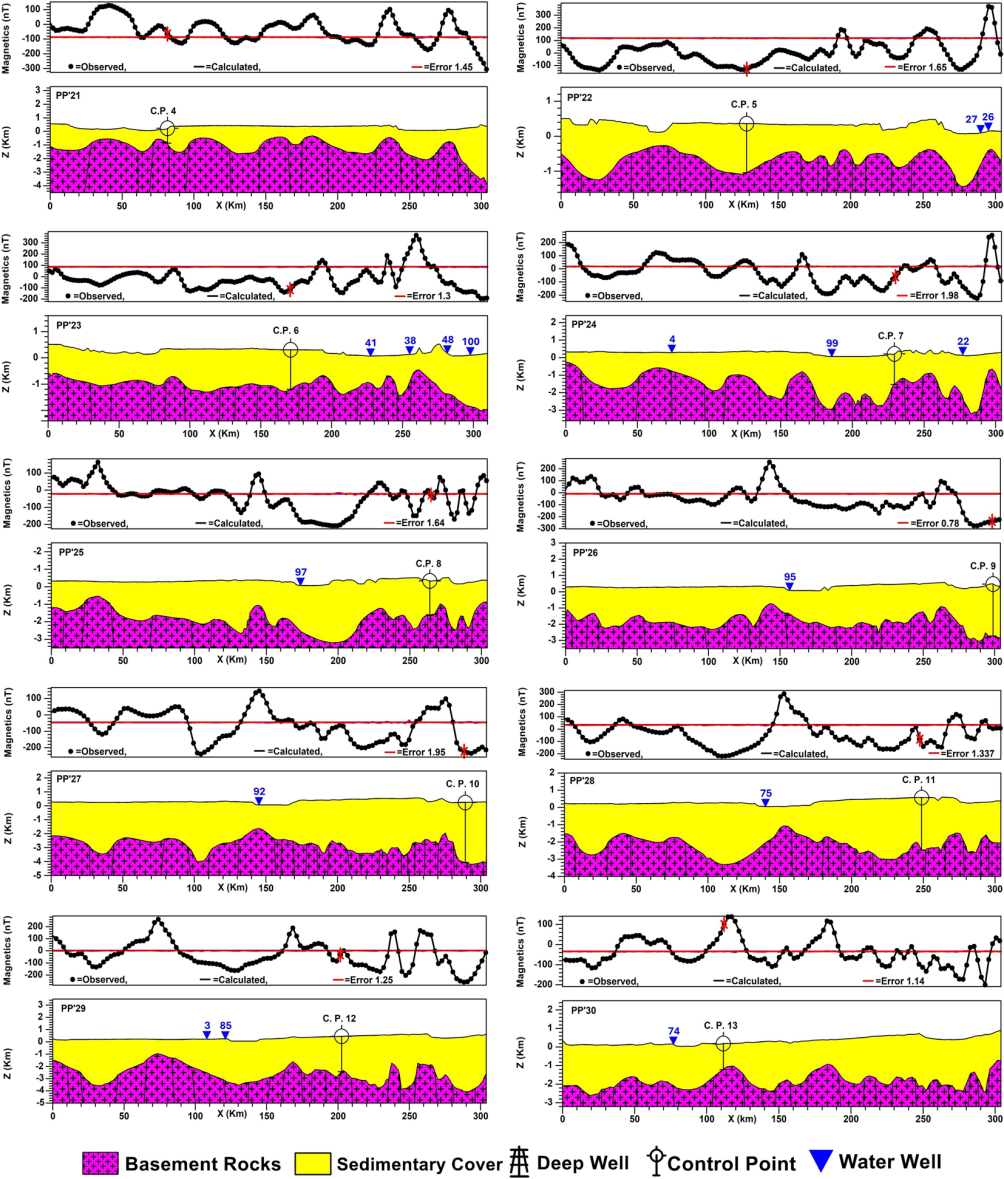
Thirty modeled 2D subsurface cross-sections with deep boreholes and water wells, for the locations of profiles and boreholes, see Fig. 6a, and for the locations of water wells, see Fig. 3f. Created by Geosoft Oasis montaj (GM-SYS 2D) (Version 8.4).

**Fig. 10:**
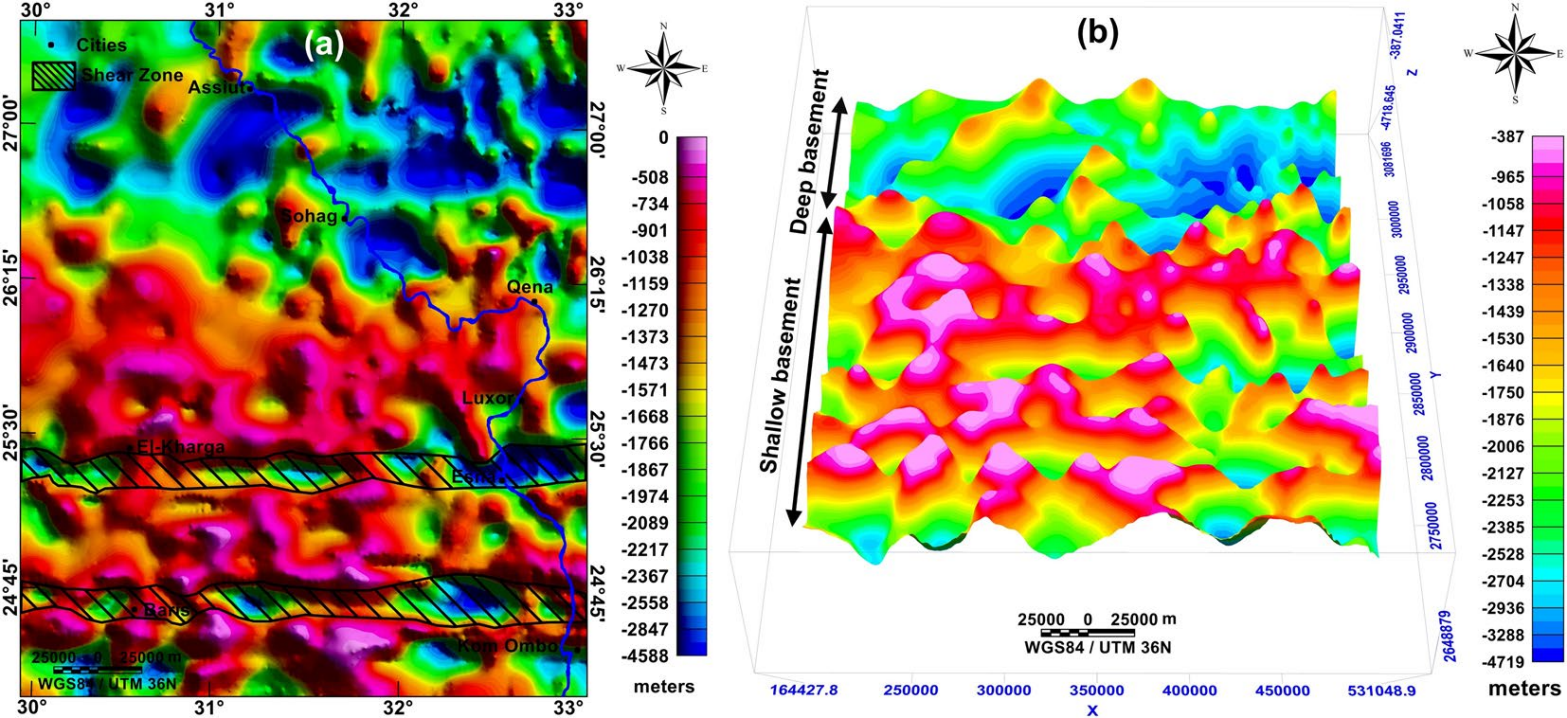
(**a**) Structure contour map of the basement relief with shear zones marked with black hatching, and (**c**) 3D-view of the depth-to-basement map. Created by Geosoft Oasis montaj (Version 8.4).

Figure 9 displays the modeled 30 profiles; the combination and interpolation of the 30 modeled 2D profiles yielded the basement relief map attributed to the msl datum. It also represents a structural map of the basement surface (Fig. 10a). The basement structure map shows prevailing shear zones in the southern parts of the study area but lacks in the northern part.

Figure 10b visualizes a 3D view of the depth-to-basement map; the depths to the basement surface range between 350 and 4700 m below the land surface. The lowest depths are in the south and increase progressively towards the north. The northern portion is the deepest, with the maximum thickness of the sedimentary cover all over the study area.

#### Groundwater recharge sources

**Fig. 11:**
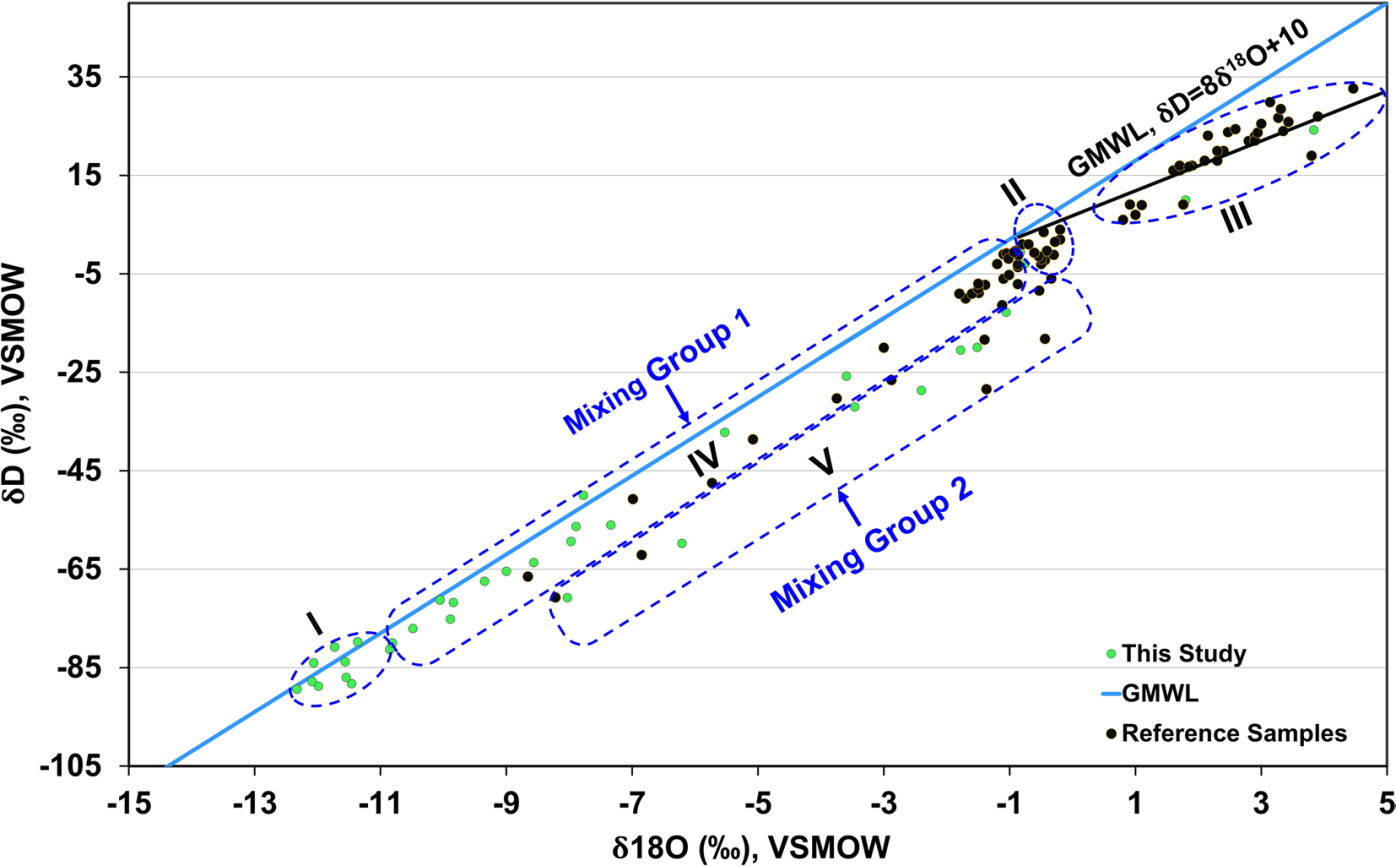
Diagram of δ^18^O** − **δD of the collected and reference samples^15–21^.

Stable isotopes values (δ^18^O, δD) of the analyzed and reference samples were depicted against the Global Meteoric Water Line (GMWL;^70^: δH = 8δO + 10) (Fig. 11). The collected samples are marked as green dots, and the black ones refer to the reference samples in the current study area from the works of^15–21^. The values for these samples range between -12.33 to 6.5‰ and -83.39 to 32.67‰ for δ^18^O and δD, respectively.

The distribution of these data includes five groups (Fig. 11): (1) Group-I includes the depleted isotopic signatures for the NAS samples in the Kharga Oasis representing fossil groundwater recharged the NAS during the wet and cool periods of the Pleistocene in agreement with the work of^27,45,65,66^, (2) Group-II samples represent the Nile water before the establishment of Aswan High Dam in 1971 (δ^18^O: − 1.3 to + 0.9‰, δD: − 3.7 to + 13.6‰^67^;), (3) Group-III includes isotopically heavier samples (δ^18^O: − 0.3 to 4.46‰, δD: − 1.14 to + 32.67‰) located along an evaporation line that represent evaporated Nile water after the establishment of Aswan High Dam^19,67^. (4, 5) Groups IV and V are respectively located along extended mixing lines between the NAS water and the old Nile water of group II and between the NAS water and the modern Nile water of group III.

According to Fig. 11, the mixing line between the NAS and shallow aquifers shows different mixing ratios for the samples lying on it and different contributions from the NAS feeding the shallow aquifers; these ratios ranged from less than 10% to 98%.

## Discussion

The fault trends detected from remote sensing data are consistent with those traced over the geologic map of Egyptian Geological Survey and Mining Authority (EGSMA) published in 1981^69^. The major surface faults in the southern and middle parts of the study area -south of latitude 26°30′N- show the various tectonic trends, the E-W, NNW, NW–SE, ENE, and NE-SW trends, showing intersection with each other. Conversely, the major surface faults in the northern parts are mainly oriented and focused on the NW-SW and NNW trends. The interpretation of aeromagnetic data revealed the trends of the major deep-seated subsurface faults. In the southern and middle parts of the study area, the main prevailing tectonic trends are E-W, NW–SE, WNW, NE-SW, NNW, and ENE. In the northern part of the study area, the deduced deep-seated faults are trending mainly in the NE-SW direction.

**Fig. 12:**
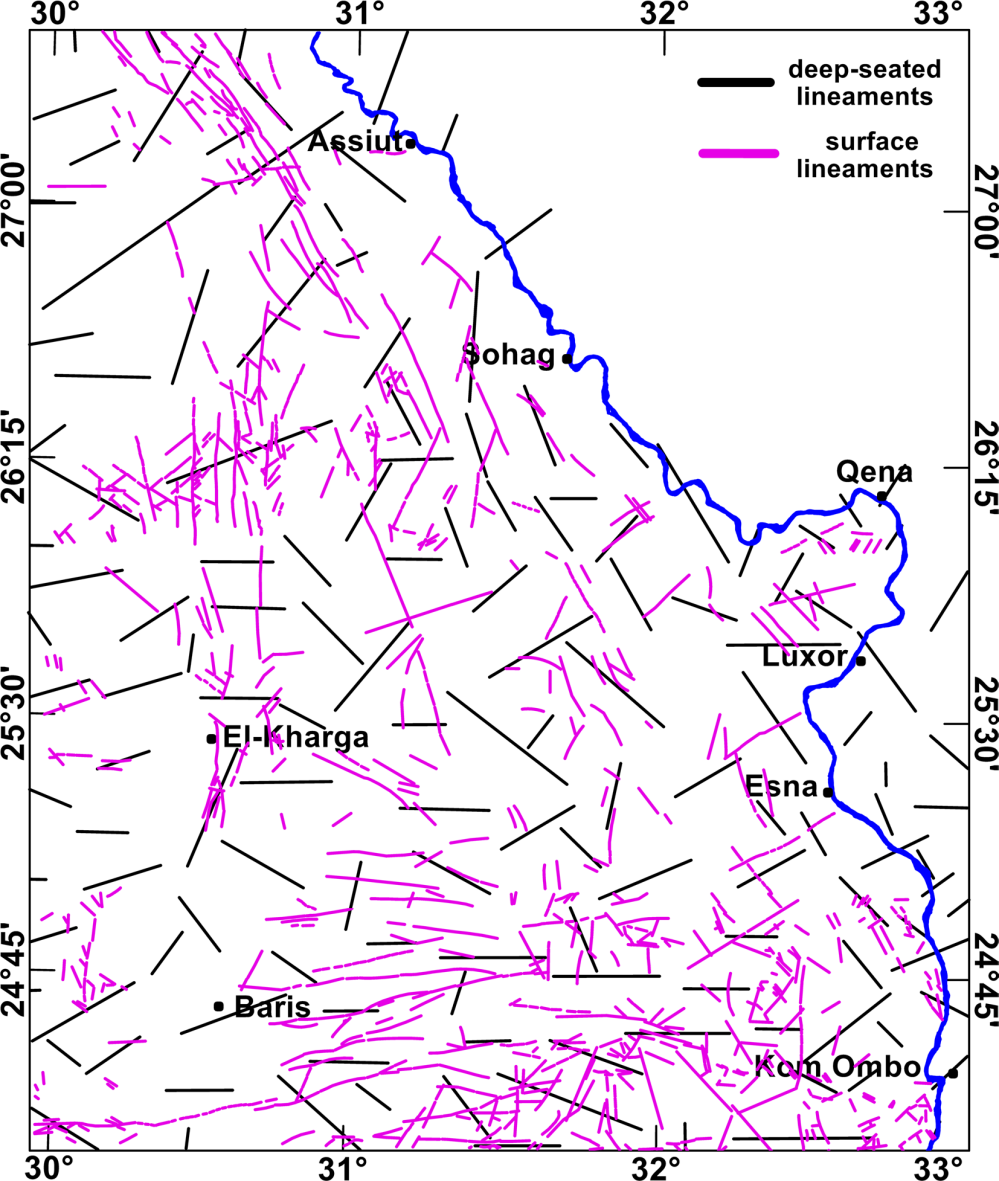
Overlay map of the deep-seated and surface lineaments in the study area. Created by Geosoft Oasis montaj (Version 8.4).

**Fig. 13:**
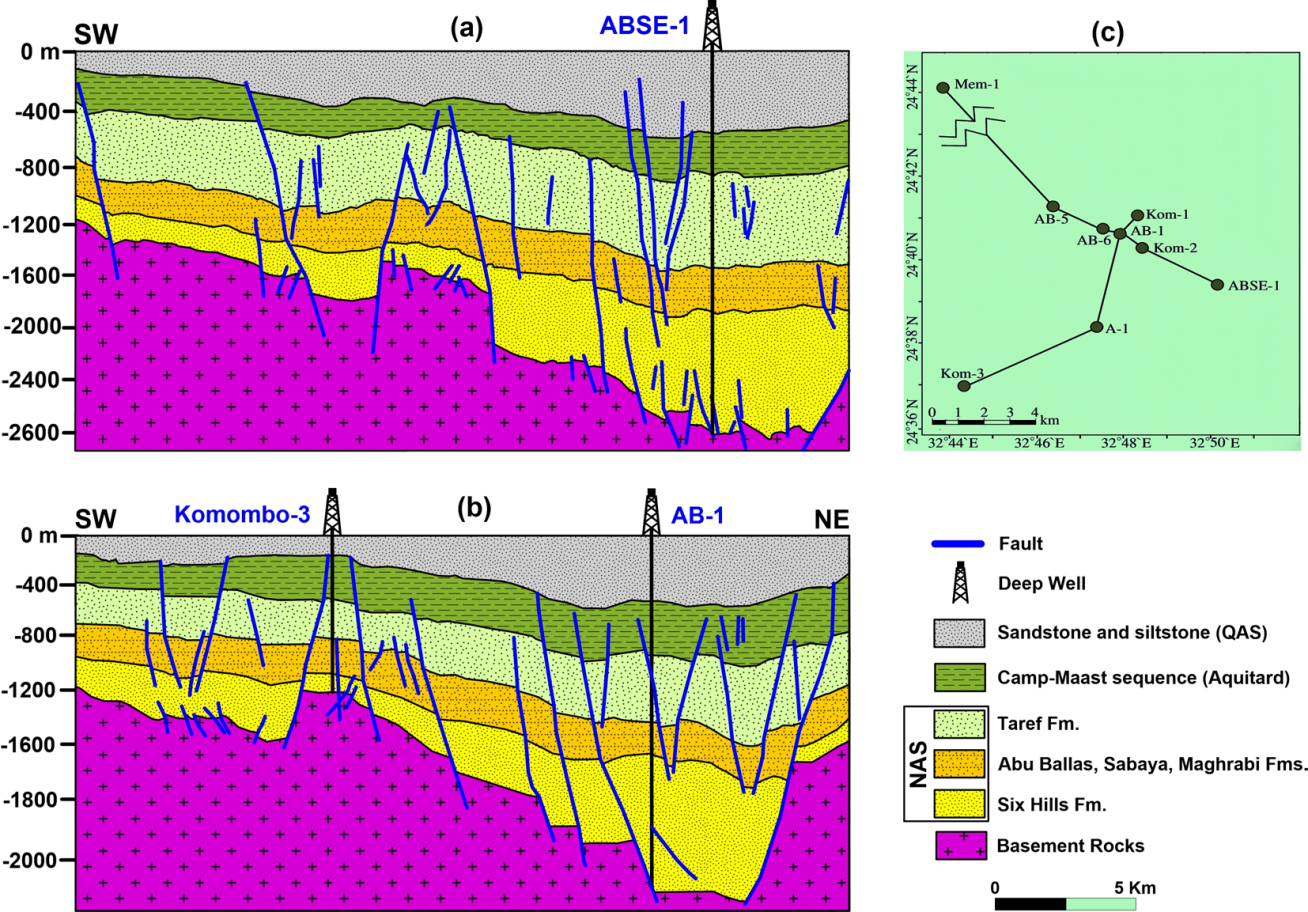
Subsurface sections illustrate fault continuity across the NAS and QAS. (**a**) and (**b**) Interpreted seismic sections in Komombo Basin , (**c**) location map of the sections. Adapted with permission of John Wiley & Sons from ‘Tectonic evolution and subsidence history of the Cretaceous basins in southern Egypt: The Komombo Basin’, Ali, M., Ali, M. Y., Abdelhady, A., Fairhead, J. D., *Basin Research*, 34(5), 2022; permission conveyed through Copyright Clearance Center, Inc. Created by Golden Software Surfer (v. 29.3).

The subsurface fault trends deduced from the STDR map are correlated to the faults obtained from the remote sensing data in the southern and middle parts (Fig. 12). Conversely, this correlation is minimal in the northern part. This points towards the upward continuity of the deep-seated and superficial faults in the southern and middle parts of the study area, unlike the northern part. This likely suggests a hydraulic connectivity between deep and shallow units in the southern and middle parts of the study area, but not in the northern part. This structural continuity is a major factor, thus supporting the hydrogeological connectivity between the NAS and the overlying aquifers in the southern and middle parts. This hypothesis is supported by the structural perspective of^39,71^, Figure 13 shows detailed interpreted seismic sections in Komombo Basin after^71^ that reflect the continuity of faults in Komombo Basin from the top of basement rocks to the entire sedimentary cover. These sections depict deep-shallow aquifer connectivity phenomena obviously at a small scale that is comparable to large-scale distribution in the Eastern Sahara.

The various tectonic trends in the southern and middle parts show considerable intersections with each other, mostly the NW, ENE, and NE trends. Many of the various tectonic trends are analogous to the prevailing rejuvenation of old Paleozoic and older structural elements during the Late Jurassic to Late Cretaceous or Early Tertiary time^72,73^.

According to the qualitative interpretation of the RTP map, the depth variation deduced by the PS curve (from less than 1 km to around 5 km) can be deduced to increase from the south to the north along the study area. The basement relief and depth to basement map displays paleohighs and basins trending in the E-W, NW–SE, WNW, NE-SW, ENE, and NNE trends. The depth to the basement rocks is increasing to the north, reflecting that the thickness of the sedimentary cover is increasing from the south to the north, from 350 to 4700 m below the land surface, respectively. The deduced shear zones in the study area represent highly deformed zones, indicating the tectonics that supported the formation of the faults in the sedimentary cover that enhance the connectivity between aquifers. This shearing is directed along the East–West direction and follows the Nubian Fault System (NFS) in the Southern Western Desert in Egypt. ^74^summarized that the NFS is a right-lateral shear zone of an intraplate type consisting of multiple E-W fault zones extending for several kilometers. They showed that the NFS is the surface feature of deep-seated basement shear zones that were formed during the augmentation of the Arabian-Nubian shield, and during the Late Cretaceous-Middle Eocene time, it was revived by rightward transpression forces. Their conclusions are consistent with the results of remote sensing and magnetic data in the present study.

**Fig. 14:**
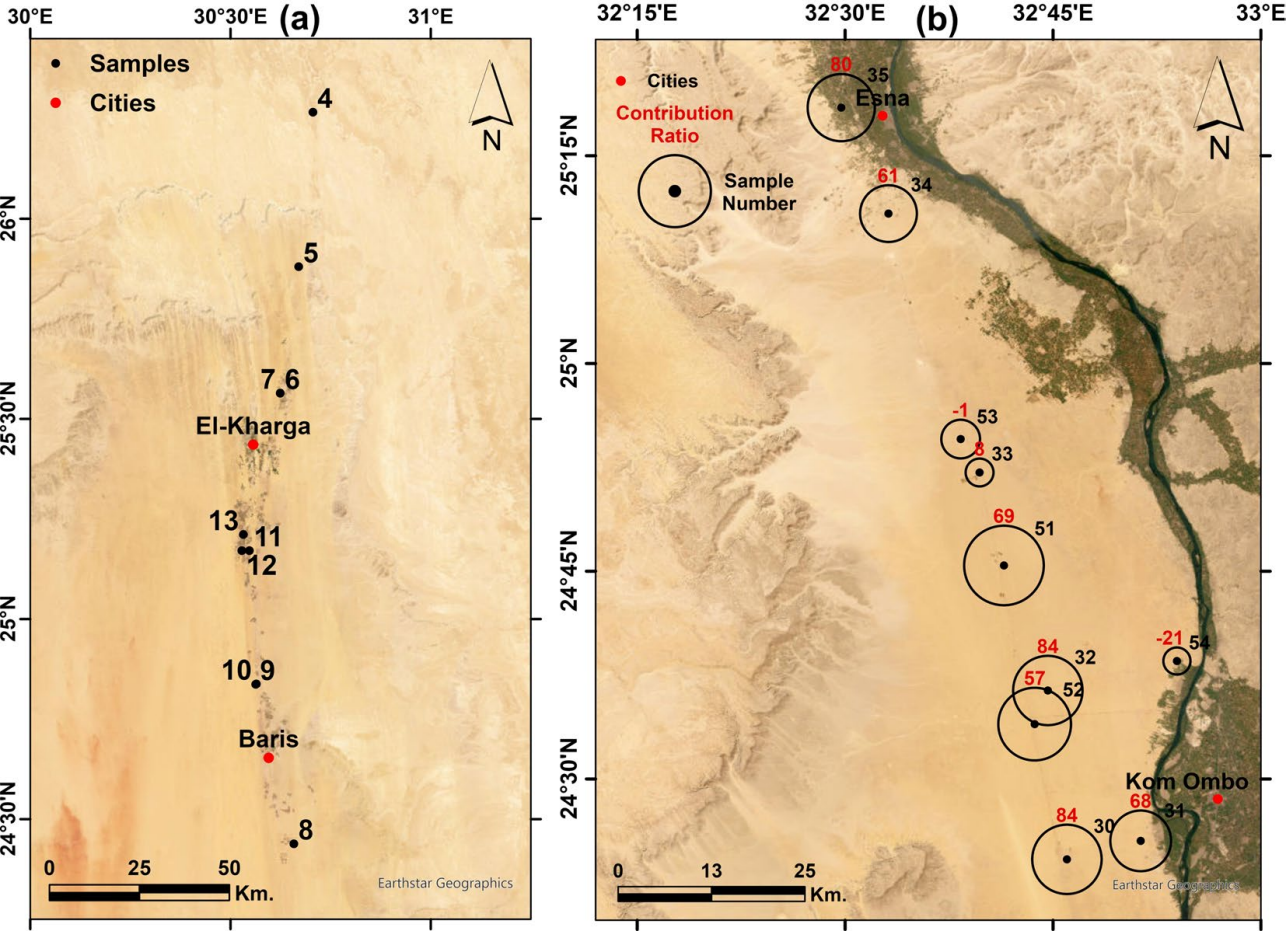
Satellite imagery showing the locations of the groundwater samples with the contribution ratios from the NAS in (**a**) El-Kharga Oasis, and (**b**) El-Gallaba Plain. Created by ArcGIS Pro (version 3.5).

After quantifying the mixing ratios between the NAS and the shallow aquifers using water isotopes, the contribution from the NAS for each sample is represented by a circle surrounding that sample, with the circle’s diameter proportional to its contribution ratio in the following regions:

The western part that contains El-Kharga Oasis (Fig. 14a), where the samples show very depleted isotopic signatures ranging between -77.05 to -89.4‰ and -10.48 to -12.33‰ for δD and δ^18^O, respectively. These samples are fossil groundwaters of the NAS that penetrated the aquifers in the Eastern Sahara in the Pleistocene pluvial times. This aquifer was formed under cooler conditions and hence its water is characterized by remarkably depleted isotopic ratios in comparison to recent precipitation^27,65,66^.

Figure 14b shows the area enclosing El-Gallaba Plain, where the samples show depleted isotopic signatures to varying degrees, indicating different contribution ratios from the NAS. The mixing ratios range between 8 to 84%, except for samples in the middle of this section (samples 37, 39) that have no contribution from the NAS. On the other hand, the remaining samples show a considerable contribution from 57 to 84%. Sample 28 shows a NAS contribution ratio of 68%. Although it represents a relatively shallow well of only 180 m total depth (TD) and depth to groundwater (DGW) of 120 m, it is only 2.5 km from the Nile Valley. Sample 35 is an artesian spring in the old, cultivated land of Esna City (named El-Ain El-Sokhna = which in English means “hot spring”). It has a natural upwelling with moderately hot temperatures, and its isotopic signature infers a contribution ratio from the NAS equal to 79%.

**Fig. 15:**
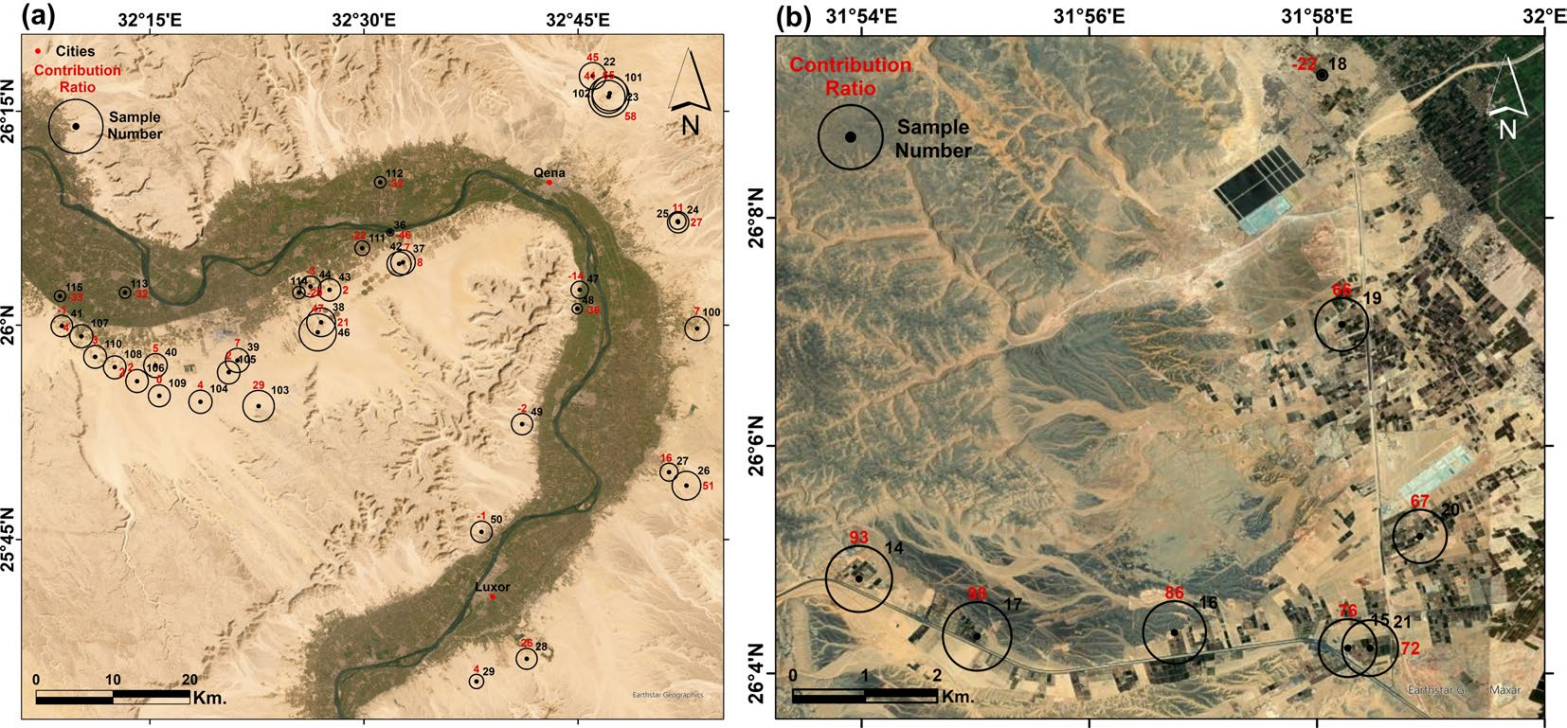
Satellite imagery showing the locations of the groundwater samples with the contribution ratios from the NAS around (**a**) the Qena Bend, and (**b**) El-Balyana area. Created by ArcGIS Pro (version 3.5).

Figure 15a shows the area around the Qena bend. In the western side of the Nile Valley, extremely nearby samples show different contribution ratios from the NAS. Sample 95 with DGW and TD of 35 and 60 m, respectively, has the highest contribution ratio from the NAS (73%), and it is 15 kms from the Nile Valley. Sample 96 with a 47% contribution ratio represents a transitional sample between the Nile Valley and adjacent land, and it is 150 and 97 for TD and DGW, respectively. The remaining samples, near or far from the valley, show low contribution ratios. Although in the current study, we mainly discuss the connectivity in the western part of the Nile Valley, we have an interest in the Eastern part of the Qena bend due to the existence of a spring (sample 23). This spring has a considerable artesian pressure and continuous discharge with a fixed rate; it has a contribution ratio of 65%. Sample 22, with only 35 m for the DGW and 70 m for the TD, has a contribution ratio of 45% despite its shallow discharge hydraulics. Both samples are 14 kilometers away from the Nile Valley.

Figure 15b shows El-Balyana area in Sohag Governorate, where we sampled wells directly along the Giza-Aswan Road. Sample 18 is the closest sample to the Nile; it has no contribution from the NAS given that it is at the rim of the Nile Valley and has shallow depth (14 and 50 m for DGW and TD, respectively). The remaining investigated samples show an increasing trend of feeding percentage from the NAS, although these samples have shallow depths ranging between 75 and 150 m only. These samples (19, 20, 21, 15, 16, 17, and 14) have high contribution ratios of 66, 67, 72, 76, 86, 98, and 93%, respectively.

**Fig. 16:**
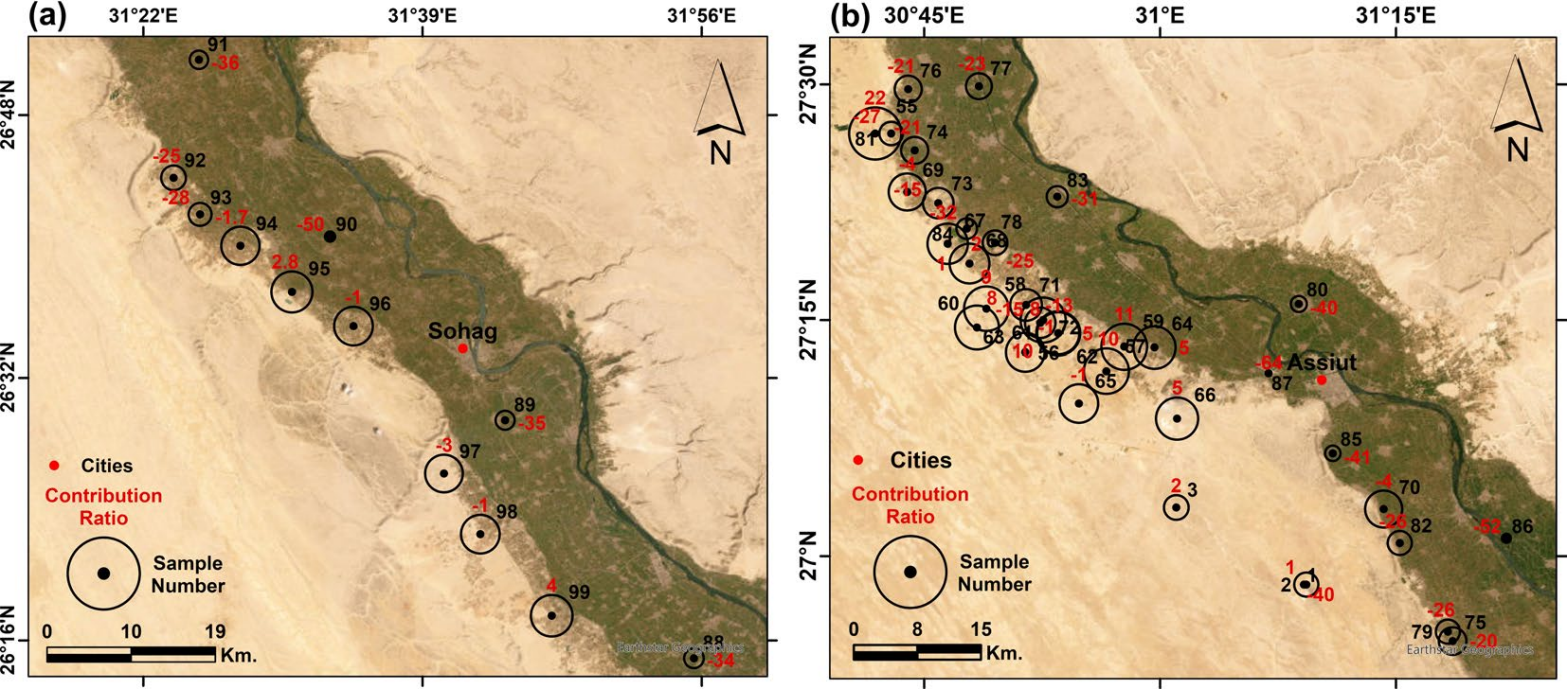
Satellite imagery showing the locations of the groundwater samples with the contribution ratios from the NAS in (**a**) Sohag area, and (**b**) Assiut area. Created by ArcGIS Pro (version 3.5).

Figure 16a displays the western side of Sohag governorate, where the samples in this area are located inside the old, cultivated land in the Nile Valley itself. The values of the stable isotopic analyses for these samples show an enrichment in the isotopic content. This means that the groundwater is recharged mainly from the Nile water.

The northern part of the study area, with a length of 90 km, represents Assiut Governorate (Fig. 16b), where there is availability of samples directly beside the Nile Valley and around it. Unlike the southern and middle areas, the NAS shows no significant contribution in this area, even for wells with a depth of 300 m (sample 3). This means that the main source of recharge is the lateral recharge from the Nile River.

It can be deduced that the feeding from the NAS in the study area has two controversial conflicting cases. The southern and middle parts may receive high to moderate recharge from the deep NAS with the high heads from the NAS. While the northern part of the study area does not seem to receive significant recharge from the deep NAS and mainly depends on the recharge from the Nile through lateral inflows.

The vertical continuity of faults and assuming faults as hydrological conduits may owe uncertainty since it is hydraulically complex and can act as conduits or barriers. In the current study, the integrated multi-proxy approach supports a conduit interpretation based on: (1) significant isotopic mass transfer (ranging between 10 and 98% NAS contribution) to shallow horizons; (2) localized artesian springs providing physical evidence of pressurized upwelling; and (3) the spatial alignment of deep-seated STDR magnetic lineaments with surface lineaments and seismic evidence in Komombo Basin (Fig. 13). This multidisciplinary evidence suggests that these structural discontinuities breach regional aquitards, enabling artesian upward leakage from the deep NAS in the southern and middle parts in the study area.

According to^20,75^, Wadi El-Assiuty (the eastern northern part of the study area) receives significant recharge from the NAS (representing 75% of incoming flow), unlike the western northern part discussed in this study (Assiut area)^20^ found that the NAS feeds the alluvial aquifer in Wadi El-Assiuty via deep-seated major subvertical NW-trending faults. They stated that the intersecting high-angle main NW and NE trending fault systems bounding alluvial sediments induce porosity and facilitate the rise of groundwater. ^76,77^ postulated that the main fault systems of the Nile Valley are NW, NE, and N-S that are initiated in the basement rocks and continue through the sedimentary cover in Wadi El-Assiuty. This structural evidence in Wadi El-Assiuty manifests the connectivity between the deep NAS and the shallow QAS based on the faults’ intersection and continuity. This scenario is analogous to the connectivity scenarios between the deep NAS and the Shallow aquifers in the southern and middle parts of the current study area in a large-scale domain.

The feeding from the deep NAS in the southern and middle parts of the study area could be related to: (1) The variation of the subsurface faults trends leading to the intersection between them (mainly NW, ENE and NE trends) facilitating the upward feeding of the NAS water, (2) The thin sedimentary cover in the southern and middle parts with varying degrees, it enhances the connectivity rather than the thick sedimentary cover in the northern part of the study area, (3) The East–West shear zones and the reactivation of the structural elements within the sedimentary cover affects the feeding dynamics (4) The upward continuity of major fault systems to the shallow aquifers playing as conduits between the NAS and shallow water horizons. While the insignificant feeding from the NAS to the shallow aquifers in the northern part of the study area could be attributed to the absence of the factors that enhance the connectivity in the southern and middle parts. In addition, the Campanian–Maastrichtian Sequence, consisting of impervious layers, probably gets thicker in the northern part, so it impedes the flow of water from the deep to the shallow horizons.

**Fig. 17:**
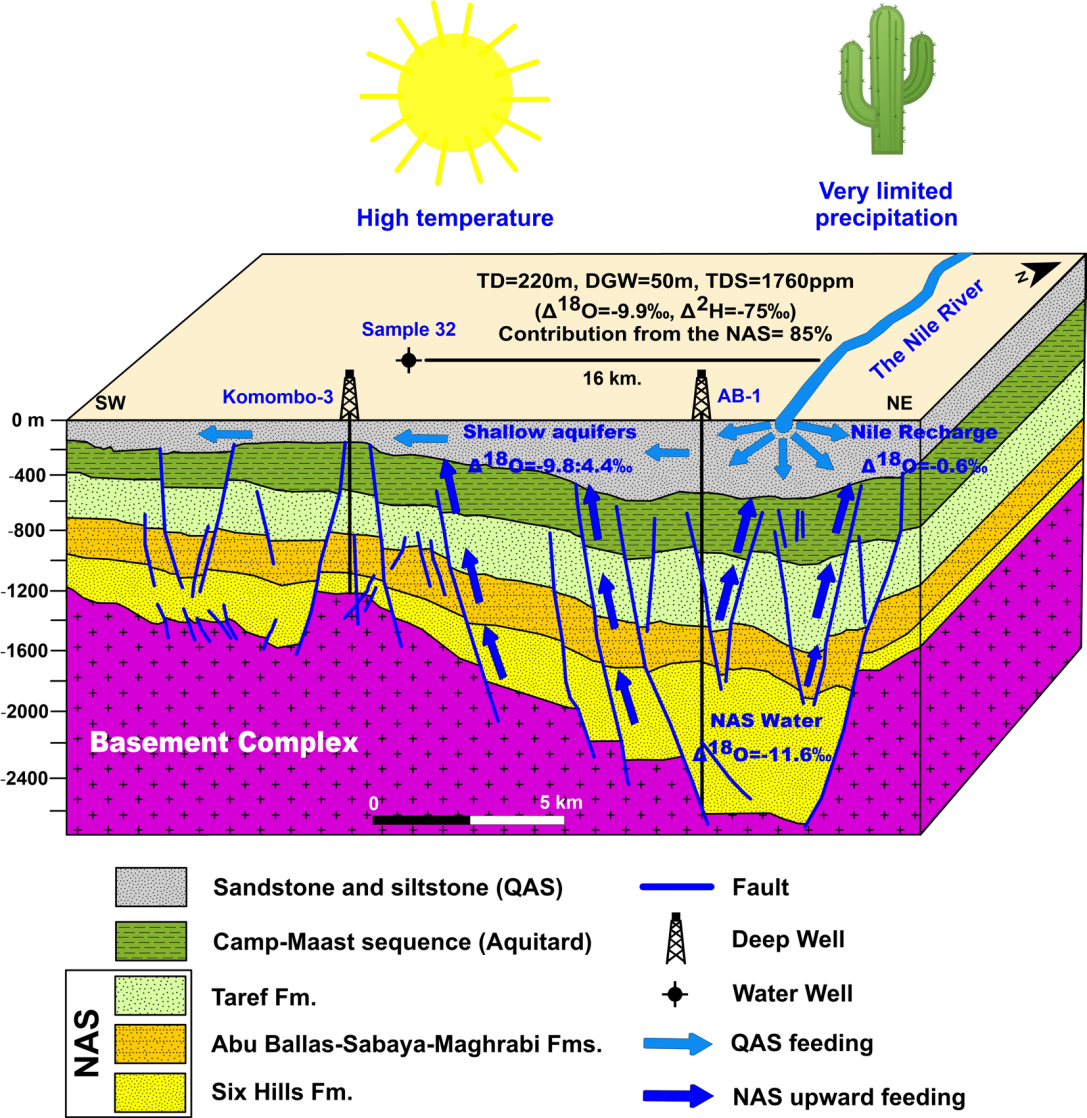
Conceptual model depicting the connectivity dynamics between deep and shallow aquifers in the study area; lithology and structure are based on the interpretation of seismic data by^71^. Lithology and structure are adapted with permission of John Wiley & Sons from ‘Tectonic evolution and subsidence history of the Cretaceous basins in southern Egypt: The Komombo Basin’, Ali, M., Ali, M. Y., Abdelhady, A., Fairhead, J. D., *Basin Research*, 34(5), 2022; permission conveyed through Copyright Clearance Center, Inc. Created by Golden Software Surfer (v. 29.3).

We developed a conceptual model to describe the groundwater flow and connectivity dynamics from the deep NAS to the shallow QAS (Fig. 17) and an example of a water sample in this study. The groundwater flows from the NAS to the shallow aquifers via major continuous faults and more likely at intersection zones between faults (most notably the NW, ENE, and NE trends) in the southern and middle parts of the study area.

The advocated model has been entirely built based on the available hydrogeological datasets in the literature^15–21^, and new isotopic data in this study, as well as the assumed mixing model, the spatial correlation between isotopic data and fault distribution and seismic data. However, some limitations might affect the model accuracy that can be summarized as follows: (1) the mixing model considered only two endmembers as the major recharge sources, given the hydroclimatic (i.e., hyper-arid conditions with less than 10 mm/yr average annual precipitation^29,66^) and geomorphologic (i.e., relatively flat-laying plateaus with no major drainage patterns^43^) settings of the Upper Nile Plateau that do not support the occurrence of additional recharge sources. However, if any additional recharge source/or sources exist would affect the mixing model and further affect the model accuracy, (2) the arithmetic averages of the utilized endmembers have been estimated based on the available isotopic analysis of the NAS and Nile water groups, and thus the variability of the isotopic composition within each group could individually affect the accuracy of the mixing values. In the meantime, the used arithmetic averages don’t lead to overestimation of the mixing ratios and the anticipated inaccuracies fractions cannot lead to big differences, and (3) the advocated model comments on a vast area based on available geophysical, remote sensing and isotope data, where the density of data vary across the area and thus the accuracy of the model would be proportional to the data availability. However, given the hyper-arid conditions of the Eastern Sahara, hydrological data is typically sparse, and yet the integrated approach presented in the present study would help to bridge the data scarcity issues, targeting the promising areas as worthy areas of exploration. In addition, future studies should apply chemical analyses to further understand the impact of mixing on the water quality and how rock-water interaction along the advocated vertical flow affects the groundwater characteristics.

## Implications of the study

**Fig. 18:**
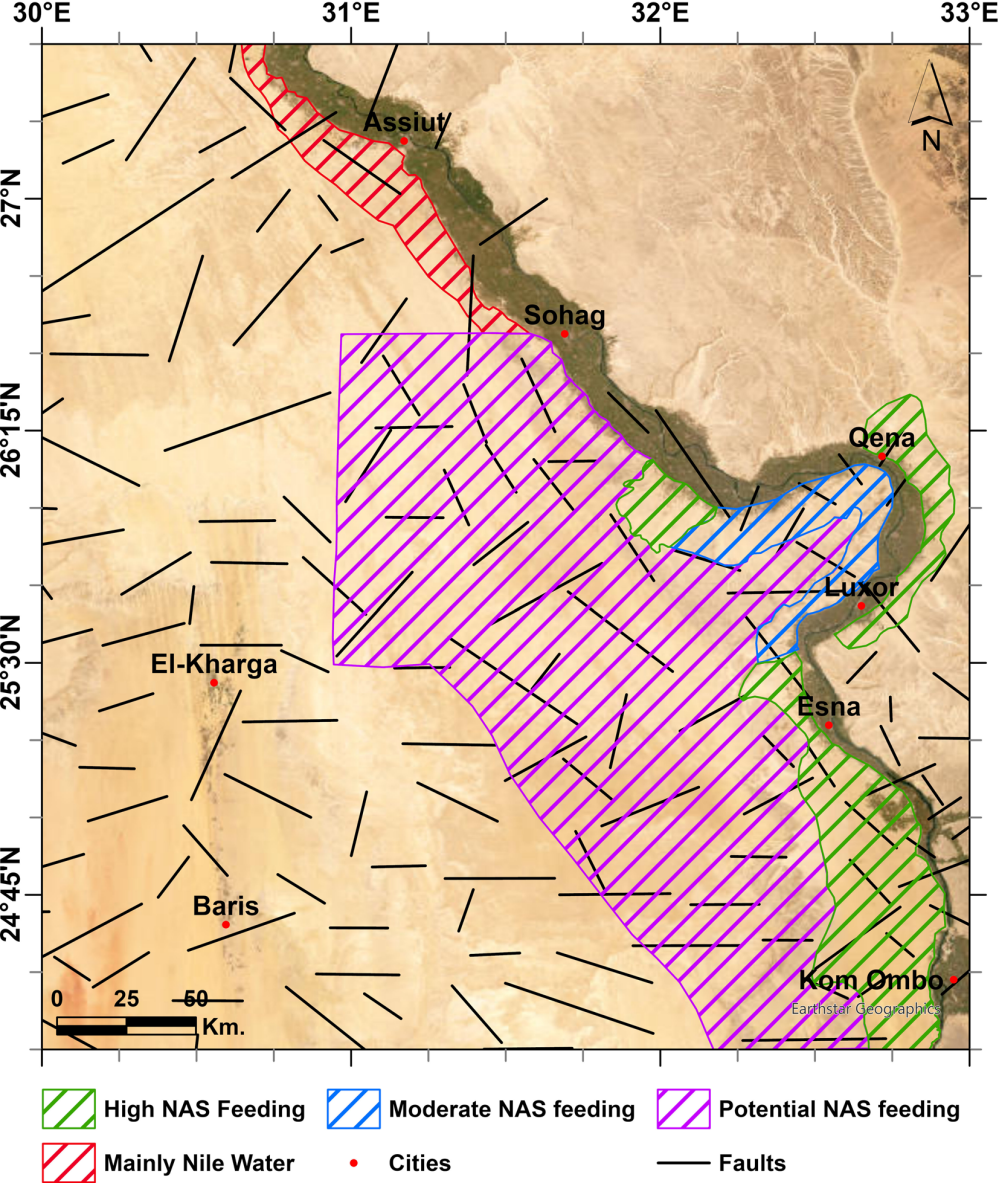
Classification of feeding from the NAS to the shallow aquifers in the study area, with the major subsurface faults. Created by ArcGIS Pro (version 3.5).

Based on the remote sensing, geophysical, and stable isotope analyses, the feeding from the deep NAS to the shallow aquifers in the study area can be classified. Figure 18 shows this classification, where the southern and middle parts around the Nile Valley may receive significant and moderate feeding from the NAS to the shallow aquifers. The northern part of the study area (Assiut) may receive limited recharge from the NAS and the shallow aquifers there are mainly recharged by the Nile water. Based on the study results and the conceptual model, including the intersection and continuity between fault systems, the relatively thin sedimentary cover, shearing and structural rejuvenation, we reasonably infer that the extensive non-explored and data-free limestone plateau (CAS) may receive considerable feeding from the deep NAS via major faults in the southern and middle parts, south of latitude 26°30′N. Several areas that may receive significant recharge from the deep NAS to the shallow aquifers coincide with key national development zones, such as the west Komombo area, which is a part of the 1.5 million feddans project. This indicates a positive potential for deep groundwater recharge from the NAS in this region.

While the study area explores new unconventional groundwater resources, which add to the strategic reserve of freshwater in Egypt, the study highlights the significance of integrated water management strategies. This may include the conjunctive use of surface water and groundwater, adapting modern technologies such as precision agriculture, and reliance on drought-resilient crops. These strategies would support the sustainable development and management of groundwater resources in the study area.

## Conclusions

This study integrates hydrogeological, stable isotopes, geophysical, and remote sensing techniques to investigate the groundwater flow dynamics and connectivity between deep and shallow aquifers in the Eastern Sahara. The study suggests that extensive areas in southern Egypt may receive considerable recharge from the NAS to the shallow aquifers along the intersection of NW, ENE, and NE structural trends on the western desert fringes of the Nile River, especially south of latitude 26°30′N. The isotopic data suggest that these intersections coincide with depleted groundwater within the shallow aquifer, indicating substantial upwelling of deep groundwater from the NAS, with contributions reaching up to 98%. Given the limited data in the limestone plateau, the advocated conceptual model, together with the deduced NW, ENE, and NE structural trends and their surface extension, may inform the most promising areas for groundwater exploration in south Egypt within the limestone plateau. These areas are expected to receive significant recharge from the NAS, as long as the NAS maintains its higher hydraulic heads and pressure, providing the groundwater necessary for agricultural development projects under an integrated water management strategy.

## Data Availability

The datasets generated or analyzed in this study are available from the corresponding authors upon reasonable request.
